# Could chronic Vardenafil administration influence the cardiovascular risk in men with type 2 diabetes mellitus?

**DOI:** 10.1371/journal.pone.0199299

**Published:** 2018-06-28

**Authors:** Daniele Santi, Michela Locaso, Antonio R. Granata, Tommaso Trenti, Laura Roli, Chiara Pacchioni, Vincenzo Rochira, Cesare Carani, Manuela Simoni

**Affiliations:** 1 Unit of Endocrinology, Department of Biomedical, Metabolic and Neural Sciences, University of Modena and Reggio Emilia, Modena, Italy; 2 Unit of Endocrinology, Department of Medical Specialties, Azienda Ospedaliero-Universitaria of Modena, Modena, Italy; 3 Department of Laboratory Medicine and Pathological Anatomy, Azienda USL of Modena, Modena, Italy; Seconda Universita degli Studi di Napoli, ITALY

## Abstract

**Introduction:**

Appropriate algorithms for the prediction of cardiovascular risk are strongly suggested in clinical practice, although still controversial. In type 2 diabetes mellitus (T2DM), the beneficial effect of phosphodiesterase (PDE)-5 inhibitors is demonstrated on endothelial function but not on the estimation of cardiovascular risk.

**Aim:**

To study whether the chronic Vardenafil administration to men with T2DM influences variables correlated with the predicted long-term cardiovascular risk calculated by different validated algorithms.

**Methods:**

Per-protocol analysis of a longitudinal, prospective, randomized, placebo-controlled, double-blind, investigator-started, clinical trial. 54 male patients affected by T2DM were assigned to study (26patients) and control-group (28patients), respectively. The study included a treatment phase (24weeks) (Vardenafil/placebo 10mg twice-daily) and a follow-up phase (24weeks). Three time points were considered: baseline(V0), end of treatment(V1) and end of the study(V2). Parameters evaluated: endothelial health-related parameters and cardiovascular risk, assessed by calculating the Framingham (coronary hart disease [CHD], myocardial infarction [MI], stroke and cardiovascular disease [CVD]), ASSIGN and CUORE equations.

**Results:**

Predicted cardiovascular risk at ten years resulted different using the three algorithms chosen, without differences between study and control groups and among visits. IL-6 was directly related to CHD, CVD and CUORE scores at V1 and with MI and STROKE at V2. Similarly, hs-CRP was directly related to CHD, MI, STROKE and CUORE only at V1 in the study group. Testosterone serum levels were inversely related to CHD and MI at V1 in study group.

**Discussion:**

The predicted cardiovascular risk is different depending on the algorithm chosen. Despite no predictive risk reduction after six months of treatment, a possible effect of Vardenafil could be hypothesized through its action on inflammation markers reduction and through restoration of normal testosterone levels.

## Introduction

Predictability of coronary heart diseases (CHD) is one of the major challenge in clinical practice [1–4]. Cardiology societies worldwide recommend the use of appropriate predictive algorithms for identification of patients at high cardiovascular risk [5–10]. The Framingham Heart study provided the first and best known example of such equations, created in 1991 thanks to a large North American population-based trial [11]. This algorithm is used to calculate the cardiovascular risk at ten years with the final aim to decide if a preventive treatment is needed [11]. A number of algorithms have been proposed in the following years, considering the population-specific risk and evaluating different large population-based cohorts. For the Italian population, in 2000, the CUORE score was created to evaluate the risk of coronary deaths [12–14]. These algorithms represent the only largely evaluated method to consider this risk in a cohort of patients.

Type 2 diabetes mellitus is one of the major risk factors of CHD [15]. Indeed, diabetes increases the long-term cardiovascular risk to the level observed in non-diabetic patients with prior myocardial infarction [16]. Diet, exercise, anti-diabetic drugs and bariatric surgery approaches have been studied for their effectiveness in reducing cardiovascular events, which is generally independent on their glucose-lowering effect [15]. In this clinical setting, despite the validation of multiple diabetes-specific and general population-based cardiovascular risk assessment models, the cardiovascular risk estimation remains approximate [17]. Moreover, the management of diabetic men does not always consider prediction of cardiovascular risk in the treatment selection.

Phosphodiesterase (PDE)-5 inhibitors are approved for erectile dysfunction and for pulmonary hypertension. These drugs act controlling the intra-cellular degradation rate of second messengers, such as cyclic guanosine monophosphate (cGMP) [18]. Consequently, PDE-5 inhibitors improve vasodilation, through the nitric oxide (NO)-dependent cGMP increase [19, 20]. This vasodilator effect has been studied at cardiovascular level, evaluating the cardiac output in case of chronic congestive heart failure [21], the remodelling action on the cardiac muscle cells, as well as the inhibition of platelet aggregation [22]. All these effects are due to the restoration of the physiologic equilibrium between mediators of vasoconstriction and vasodilation, inhibiting the catabolism of cGMP and enhancing NO levels [23]. These endothelial function improvements could reduce atherogenesis and vascular complications of diabetes mellitus [23]. Despite an increasing evidence of these beneficial effects [24], no studies so far evaluated whether PDE-5 inhibitors administration influences the long-term cardiovascular risk prediction in diabetic patients.

In this study we assessed whether the chronic Vardenafil administration to men with type 2 diabetes mellitus could reduce the long-term estimated cardiovascular risk, using validated algorithms. Since no specific algorithm to predict cardiovascular risk in diabetic men is commonly accepted as the reference method so far, three different equations were used and compared.

## Materials and methods

### Study design

**Fig 1:**
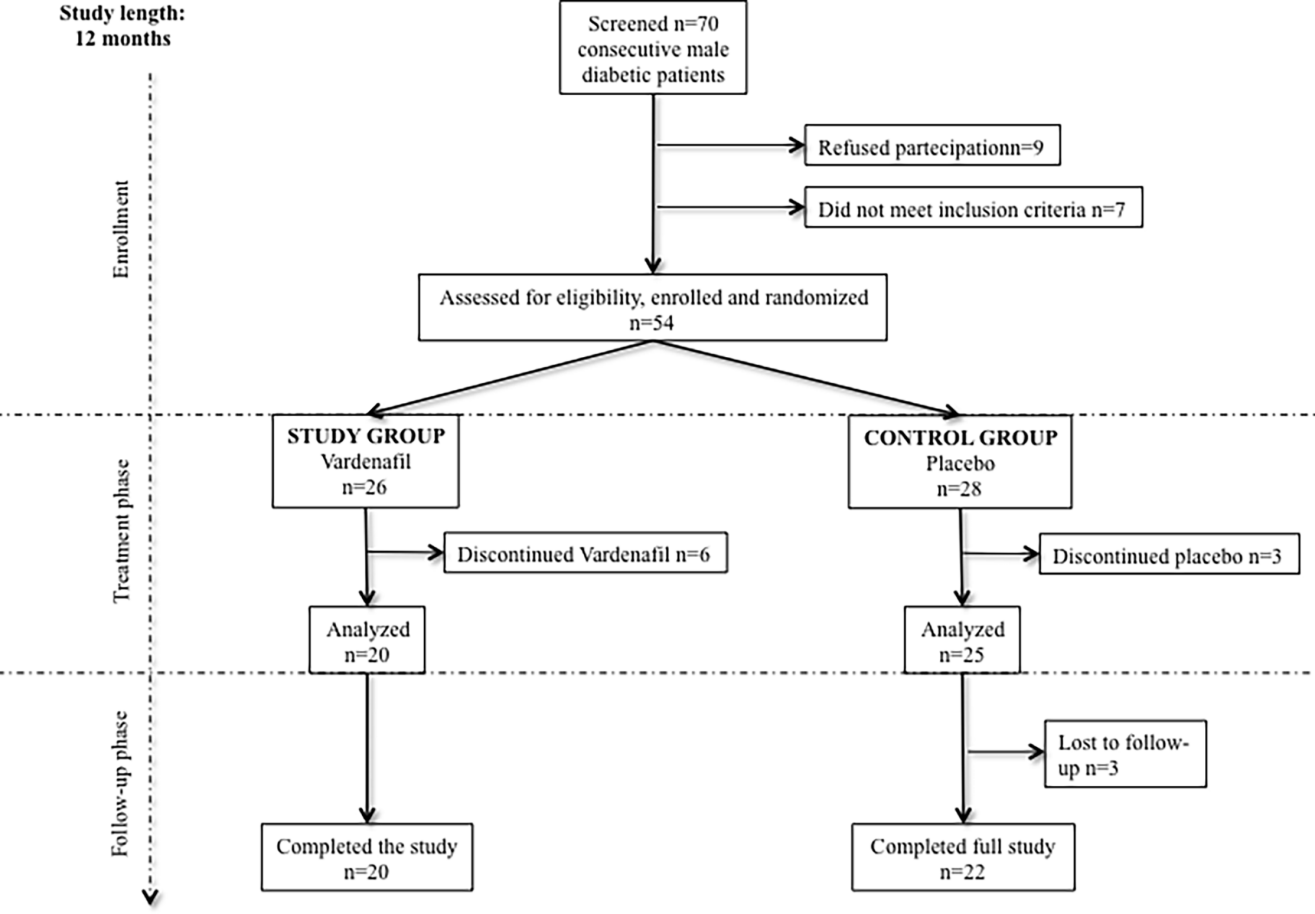
Study flow chart.

This is a secondary, retrospective, analysis of a recent longitudinal, prospective, randomized, placebo-controlled, double-blind, investigator-started, clinical trial described in detail earlier [25]. Briefly, we enrolled 54 patients with diagnosis of type 2 diabetes mellitus made within 5 years before enrollment, and with erectile dysfunction but no previous use of PDE-5 inhibitors (Fig 1). The study lasted from 2008 to 2014. Patients were randomized using the permuted block method and assigned to the study or control group, in which Vardenafil (10 mg twice daily) or placebo were administered, respectively [25]. Patients were followed for one year, considering six months of therapy and six months of treatment withdrawal. During the year of trial, each patient was evaluated ten times, 8 during the treatment phase and 2 during the follow-up [25]. During each visit, a blood sample was taken after an overnight fast and biochemical and endothelial health-related parameters were evaluated and reported in our previous paper [25]. Here, we used biochemical parameters and clinical history of the patients to calculate the cardiovascular risk using three different validated algorithms. In particular, using a per-protocol analysis, we considered three visits carried out six months away: baseline (V0), end of treatment (V1) and end of the study (V2).

### Cardiovascular risk assessment

The cardiovascular risk was calculated using three appropriate validated algorithms available in the literature. First, the Framingham algorithm [11] was used, which considers the four following outcome risks: CHD, myocardial infarction (MI), stroke and cardiovascular disease (CVD). Second, the ASSIGN equation and the CUORE score were used as alternative tools to cardiovascular risk calculation. The ASSIGN risk score derives from the Scottish Heart Health Extended cohort study, which includes several cohorts with more than 6000 men aged between 30 and 74 years [26]. The CUORE risk score [27–29] derives from a study including a combination of 11 Italian cohorts with more than 6800 men aged between 35 and 69 years [14].

Overall, these three tools predict the cardiovascular risk considering age, sex, total cholesterol, HDL, diabetes mellitus, smoking habit and systolic pressure.

### Cardiovascular and biochemical parameters

The calculated cardiovascular risk was evaluated in association with markers of endothelial health. In particular, we considered ultrasonography parameters, such as flow mediated dilation (FMD) [30, 31], and biochemical markers, such as interleukin (IL)-6, intercellular adhesion molecule (ICAM)-1, vascular cell adhesion molecule (VCAM)-1, high sensitivity C-reactive protein (hs-CRP), endothelin (ET)-1 and testosterone serum levels.

IL-6 was measured on plasma samples by the Human IL-6 Quantikine ELISA Kit (R&D Systems Europe, Ltd. Abingdon OX14 3NB, UK). ICAM-1 and VCAM-1 were measured on serum samples, diluted 1:2, using the bead-based multiplex assay for Luminex platform (R&D Systems Inc kit code LXSAH, Minneapolis, US). Hs-CRP and ET-1 were measured with the BN™ II System instrument (Siemens) through a specific automated nephelometric assay (CardioPhase® hsCRP, Dada Behring Inc. Newark, DE 19714 US). Testosterone was analyzed by liquid-chromatography, tandem mass-spectometry (LC/MS-MS) (Shimadzu Nexera UHPLC with Shimadzu LCMS-8050 triple quadrupole) with the Pelkin Elmer kit (Wallac OY, Turku, Finland), using serum samples obtained at each visit. Other routine assays were performed using commercially available kits.

### Ethics

The local Ethics Committee of the University of Modena and Reggio Emilia, Italy, approved the study protocol. Patient informed consent was obtained and patients were appropriately insured. The study was registered according to European study registration rules (EudraCT: 2009-014137-25), and on ClinicalTrials.gov (Identification number NCT02219646)

### Statistical analysis

Statistical analysis was performed through the “per protocol” method. According to the not-normal distribution of variables, evaluated at Kolmogorov-Smirnov test, the differences of risk scores were evaluated comparing study and control groups using Mann-Whitney test at each visit. The score change during the study protocol was evaluated through Wilcoxon-rank test in both groups. Moreover, at each visit, the risk change compared to previous visit was calculated. Finally, each risk was evaluated after adjustment for blood pressure and total, HDL and LDL cholesterol. The correlation among the risk scores and other parameters was evaluated using Spearman’s Rho.

Finally, multivariate analyses were performed to detect possible predictors of cardiovascular risks. Thus, stepwise, linear, multiple regression analyses were performed using each risk score as dependent variable and age, body mass index, glucose, glycated haemoglobin, microalbuminuria, hs-CRP, IL-6, ET-1, ICAM-1, VCAM-1, fibrinogen, FMD and testosterone as independent variables. Each independent variable entered the analysis step by step. In these analyses lipid profile and blood pressures have not been considered as dependent variables, since they are included in the algorithms used for cardiovascular risk calculation. All multiple regression analyses were performed in the study group.

Statistical analysis was performed using the ‘Statistical Package for the Social Sciences’ software for Macintosh (version 21.0; SPSS Inc., Chicago, IL). For all comparisons, p values < 0.05 were considered statistically significant.

## Results

Fifty-four diabetic men were enrolled, 26 in the study and 28 in the control group. Excluding drop-outs, 20 patients in the study and 25 in the control group were finally evaluated. Patients enrolled were similar at baseline for each parameter investigated, as previously reported (Table 1) [25].

**Table 1:** Baseline biochemical characteristics of enrolled patients, evaluated by per protocol analysis.

	Normal range	Overall	Vardenafil	Placebo	P-value
*Number of patients*	-	45	20	25	-
*IIEF-15 –* *Erectile function domain*		17.17±7.65	16.62±7.90	17.68±7.51	0.614
*Glycemia (g/dL)*	70–110	138.61±41.21	141.65±47.17	135.79±35.44	0.606
*HbA1c (%)*	4–6	7.04±1.04	7.09±1.07	6.99±1.02	0.738
*Total cholesterol (mg/dL)*	<200	175.52±36.53	170.38±34.28	180.29±38.50	0.324
*HDL cholesterol (mg/dL)*	>39	42.43±8.23	42.35±7.98	42.50±8.60	0.946
*Triglycerides (mg/dL)*	<180	140.93±66.93	131.00±57.73	150.14±74.33	0.298
*LDL cholesterol (mg/dL)*	<115	104.91±33.44	101.84±30.67	107.76±36.15	0.521
*FMD (%)*	>7	7.38±4.55	6.83±3.89	7.87±5.09	0.308

The basal cardiovascular risk at ten years was different using the three algorithms chosen (p = 0.004), ranging from 12.10±9.30% for the CUORE equation, to 14.86±7.55% for the Framingham CHD score, to 21.48±7.48% for the ASSIGN equation. At V0, no differences between study and control groups were found for CHD, MI, stroke, CVD, ASSIGN and CUORE scores (Table 2). Similarly, no differences were found at V1 (CHD p = 0.405, MI p = 0.372, stroke p = 0.759, CVD p = 0.407, ASSIGN p = 0.708 and CUORE p = 0.668) and V2 (CHD p = 0.455, MI p = 0.327, stroke p = 0.425, CVD p = 0.336, ASSIGN p = 0.809 and CUORE p = 0.661). No differences among visits were found, considering separately the study (CHD p = 0.767, MI p = 0.755, stroke p = 0.903, CVD p = 0.862, ASSIGN p = 0.417 and CUORE p = 0.962) and control (CHD p = 0.806, MI p = 0.849, stroke p = 0.981, CVD p = 0.951, ASSIGN p = 0.194 and CUORE p = 0.999) group. Similarly, no changes were observed after adjustment for cholesterol and blood pressure.

**Table 2:** Basal cardiovascular risk, calculated using the three different equations.

	CHD (%)	MI (%)	STROKE (%)	CVD (%)	ASSIGN (%)	CUORE (%)
**Study group**	14.22±7.67	8.49±6.43	4.50±4.19	22.75±12.10	19.19±6.90	11.82±9.12
**Control group**	11.51±7.44	9.66±6.05	4.89±4.19	24.85±11.65	23.07±8.07	12.39±9.49
*p-value*	*0*.*536*	*0*.*497*	*0*.*734*	*0*.*524*	*0*.*767*	*0*.*824*

Footnotes: CHD: coronary heart disease, MI: myocardial infarction, CVD cardiovascular disease

Considering the change of each cardiovascular risk estimation from V0 to V1 and from V1 to V2, no differences were found between study and control group.

### Endothelial health-related parameters

We previously demonstrated that chronic Vardenafil treatment significantly decreases IL-6 serum levels [25]. Here, we found that IL-6 was not related to cardiovascular risk estimated at baseline. However, IL-6 was directly related to CHD, CVD and CUORE scores at V1 (Table 3). These correlations remained statistically significant after treatment withdrawal (V2) for MI and STROKE (Table 3).

**Table 3:** Correlation between IL-6 serum levels and cardiovascular risk scores.

		Study group			Control group		
		V0 (n = 26)	V1 (n = 20)	V2 (n = 20)	V0 (n = 28)	V1 (n = 25)	V2 (n = 23)
**Overall cohort**							
CHD	Coefficient	0.180	0.432	0.432	0.213	0.186	0.327
	p-value	0.449	**0.027**	**0.047**	0.285	0.374	0.128
MI	Coefficient	0.146	0.323	0.475	0.148	0.158	0.379
	p-value	0.539	0.108	**0.035**	0.462	0.449	0.074
STROKE	Coefficient	0.207	0.382	0.486	0.339	0.129	0.335
	p-value	0.380	0.054	**0.030**	0.082	0.538	0.119
CVD	Coefficient	0.194	0.394	0.508	0.234	0.126	0.389
	p-value	0.412	**0.047**	**0.022**	0.240	0.549	0.066
ASSIGN	Coefficient	0.032	0.335	0.197	-0.052	0.036	0.259
	p-value	0.894	0.095	0.405	0.797	0.866	0.233
CUORE	Coefficient	0.237	0.454	0.601	0.344	0.198	0.418
	p-value	0.314	**0.020**	**0.005**	0.079	0.344	0.097

Footnotes: CHD: coronary heart disease, MI: myocardial infarction, CVD cardiovascular disease

Hs-CRP was not related to cardiovascular risk scores at V0 in both study and control groups (Table 4). However, a significant direct relationship was found at V1 between hs-CRP and CHD, MI, STROKE, CVD and CUORE only in the study group (Table 4). The significant correlation between hs-CRP and CHD remained at V2 (Table 4). Interestingly, the direct correlation between hs-CRP and CHD was found also in the control group at V1 and V2 (Table 4).

**Table 4:** Correlation between hs-CRP serum levels and cardiovascular risk scores.

		Study group			Control group		
		V0 (n = 26)	V1 (n = 20)	V2 (n = 20)	V0 (n = 28)	V1 (n = 25)	V2 (n = 23)
**Overall cohort**							
CHD	Coefficient	0.396	0.465	0.440	-0.054	0.425	0.433
	p-value	0.085	**0.039**	**0.043**	0.788	**0.034**	**0.039**
MI	Coefficient	0.393	0.468	0.322	-0.059	0.392	0.390
	p-value	0.091	**0.037**	0.167	0.771	0.053	0.066
STROKE	Coefficient	0.249	0.465	0.131	-0.159	0.110	0.334
	p-value	0.220	**0.039**	0.583	0.428	0.601	0.119
CVD	Coefficient	0.364	0.517	0.311	-0.087	0.329	0.321
	p-value	0.068	**0.020**	0.182	0.667	0.108	0.075
ASSIGN	Coefficient	0.333	0.294	0.396	0.287	0.276	0.321
	p-value	0.096	0.209	0.084	0.146	0.181	0.136
CUORE	Coefficient	0.354	0.527	0.350	-0.074	0.225	0.339
	p-value	0.076	**0.017**	0.130	0.714	0.280	0.136

Footnotes: CHD: coronary heart disease, MI: myocardial infarction, CVD cardiovascular disease

ET-1, ICAM-1, VCAM-1 and FMD did not correlate with any of the cardiovascular risk estimations considered, neither in the study, nor in the control group. Similarly, no correlations were found between cardiovascular risk estimation and glycemia-related parameters, such as serum glucose levels, glycated haemoglobin and microalbuminuria. Finally, the score at erectile function domain of international index of erectile function (IIEF)-15 did not correlate with estimated cardiovascular risk scores in any group.

### Gonadal status

Testosterone serum levels were not related to any of the estimated cardiovascular risk scores at V0 in either groups. However, at V1, a significant, inverse relationship between testosterone and CHD and MI was found only in the study group (Table 5).

**Table 5:** Correlations between testosterone serum levels and cardiovascular risk scores.

		Study group			Control group		
**Overall cohort**							
		V0 (n = 26)	V1 (n = 20)	V2 (n = 20)	V0 (n = 28)	V1 (n = 25)	V2 (n = 23)
CHD	Coefficient	-0.040	-0.413	-0.133	0.157	-0.320	0.051
	p-value	0.842	**0.040**	0.544	0.445	0.169	0.830
MI	Coefficient	-0.053	-0.397	-0.134	0.072	-0.346	-0.018
	p-value	0.792	**0.049**	0.541	0.725	0.135	0.940
STROKE	Coefficient	0.002	-0.318	-0.159	0.130	-0.096	-0.114
	p-value	0.993	0.121	0.468	0.527	0.686	0.631
CVD	Coefficient	-0.020	-0.344	-0.110	0.091	-0.284	0.001
	p-value	0.921	0.092	0.618	0.657	0.225	0.999
ASSIGN	Coefficient	-0.019	-0.288	-0.058	-0.010	-0.352	0.015
	p-value	0.926	0.163	0.792	0.961	0.128	0.950
CUORE	Coefficient	-0.118	-0.369	-0.173	0.102	-0.087	0.006
	p-value	0.556	0.069	0.430	0.622	0.715	0.980
**Hypogonadal men**							
		V0 (n = 7)	V1 (n = 6)	V2 (n = 6)	V0 (n = 6)	V1 (n = 4)	V2 (n = 4)
CHD	Coefficient	0.250	-0.829	-0.257	-0.281	0.034	-0.342
	p-value	0.589	**0.042**	0.623	0.647	0.966	0.778
MI	Coefficient	0.250	-0.829	-0.371	-0.274	-0.049	-0.401
	p-value	0.589	**0.042**	0.468	0.655	0.951	0.738
STROKE	Coefficient	0.071	-0.314	-0.257	-0.646	0.090	-0.094
	p-value	0.879	0.544	0.623	0.239	0.910	0.940
CVD	Coefficient	0.143	-0.829	-0.371	-0.376	0.015	-0.294
	p-value	0.760	**0.042**	0.468	0.533	0.985	0.810
ASSIGN	Coefficient	-0.071	-0.943	-0.486	0.028	0.378	-0.158
	p-value	0.879	**0.005**	0.329	0.965	0.622	0.899
CUORE	Coefficient	0.286	-0.314	-0.257	-0.632	0.312	-0.023
	p-value	0.535	0.544	0.623	0.252	0.688	0.985

Footnotes: CHD: coronary heart disease, MI: myocardial infarction, CVD cardiovascular disease

Considering gonadal status, we previously demonstrated that 13 of patients enrolled (24%) were hypogonadal at baseline, according to the cut-off limit (10.4 nmol/L) proposed by the Endocrine Society [32]. In these men, total testosterone serum levels significantly improved in the study compared to the control group and across visits [25]. Here, we further strengthen the association between testosterone and cardiovascular risk. Indeed, considering 7 hypogonadal men treated with Vardenafil, the inverse relationship at V1 was confirmed between testosterone and CHD, MI, CVD and ASSIGN (Table 5). On the contrary, no relationship was found in the non-hypogonadal men (Table 5).

### Multivariate analyses

By multivariate analyses, some significant results were detected. First, considering CHD as dependent variable, the following equation (p = 0.007) was found: CHD = 12.873+6.178*(hs-CRP). hs-CRP entered also in the second model (p = 0.009), in which the ASSIGN score was used as dependent variable: ASSIGN = 9.355+39–019*(hs-CRP). These results pinpoint variables are correlated with these parameters which enter in the prediction of cardiovascular risk, although a direct relationship was not previously pointed out. On the other hand, considering the CUORE score as dependent variable, IL-6 entered the model (p = 0.009) as follows: CUORE = 3.454+2560*(IL-6).

## Discussion

Here, we retrospectively evaluated the effect of chronic Vardenafil administration on the estimation of cardiovascular risk in diabetic men. To the best of our knowledge, this is the first trial giving the opportunity of evaluating the effect of PDE-5 inhibitors on prediction of cardiovascular risk in patients with type 2 diabetes mellitus. In spite of the beneficial effect of chronic Vardenafil administration on several endothelial health-related parameters [25], no significant change in the prediction of cardiovascular risk was found using three different algorithms. Of the variables used in these algorhytms, neither cholesterol, nor blood pressure are directly influenced by Vardenafil administration, as previously demonstrated [25].

The scientific literature demonstrates beneficial effects of PDE-5 inhibitors on cardiac remodelling [33], symptoms of coronary artery diseases [34] and hemodynamic parameters [35]. These effects were evaluated in specific clinical conditions, e.g. heart failure, myocardial infarction, stroke and hypertension [36]. In diabetes mellitus only endothelial health-related parameters were evaluated after PDE-5 inhibitors treatment [24]. We previously confirmed the beneficial effect of chronic Vardenafil administration on endothelial parameters, suggested by a IL-6 reduction and a FMD increase [25]. Whether this beneficial effect could influence the long-term cardiovascular risk in type 2 diabetes mellitus is not known. Here we do not test the cardiovascular risk of our patients but whether the prediction of this risk changes under chronic PDE-5 inhibitor treatment for six months. This parameter does not change. However, the improvement of endothelial health-related parameters resulting from chronic Vardenafil administration opens the possibility of a stronger effect of this treatment of the predicted cardiovascular risk using longer treatment duration.

A direct relationship between hs-CRP and cardiovascular risk scores is found in study group at V1, although hs-CRP did not change over time and after Vardenafil administration, as previously reported [25]. This result suggests that hs-CRP could have an important role on the parameters used in the prediction of cardiovascular risk. On the contrary, other endothelial health-related parameters, which are strongly influenced by Vardenafil administration (e.g. FMD) [25], are not related to the estimated cardiovascular risk scores. These relationships suggest that cardiovascular risk predicted by validated tools seems to be mostly related to inflammation markers, rather than to vasodilator parameters. As a confirmation, multivariate analyses highlight the hs-CRP and IL-6 role in the prediction of cardiovascular risk. Thus, since the PDE-5 inhibitors anti-inflammatory effect was proven by previous results, we might expect them to exert a protective role on cardiovascular risk provided the treatment is sufficiently long. However, the clinical use of these drugs for this purpose is not indicated so far and properly designed, longer and well-powered clinical trials are needed to better understand long-term survival of these patients after longer chronic treatment. In this context, the role of sphingosine-1-phosphate is widely proposed and a possible combined effect with PDE-5 inhibitors could be evaluated in the near future [37, 38].

Finally, cardiovascular risk predicted by validated algorithms seems to be influenced by total testosterone serum levels. In particular, CHD and MI are inversely related to testosterone after Vardenafil administration. This correlation is further confirmed considering only diabetic men with hypogonadism at baseline. Since chronic Vardenafil administration improves testosterone serum levels in hypogonadal diabetic men [25], this might be another way by which gonadal function improvement results in a beneficial effect on cardiovascular health. Therefore, the overall protective role of PDE-5 inhibitors on cardiovascular health might be mediated by both inflammatory markers decrease and gonadal function improvement.

A large number of cardiovascular risk calculators have been developed and validated so far, the first and widely used tool being represented by the Framingham score [11], which however, suffers of a number of pitfalls. Indeed, it is well demonstrated that the use of this calculator for deciding whether preventive therapy is needed or not could lead to under-treatment with a consequent socio-economic burden increase [39]. Thus, other equations have been developed and validated in different population-based cohorts. Although these algorithms should provide overlapping scores, we found different percentages of the estimated cardiovascular risk at ten years considering the same group of patients, ranging from 12% for the CUORE score, to 22% for the ASSIGN equation. Thus, our results confirm the discrepancy in the risk prediction obtained by different algorithms and how to generalize the risk score obtained by these calculators remains the main challenge in this field. Moreover, the clinical use of these algorithms must consider the characteristics of the population in which the tool has been validated. Admittedly, our study, which was not designed to assess the cardiovascular risk and solve these discrepancies, is bases on a low number of patients enrolled and relatively short-term treatment therapy length to investigate appropriately the effects of PDE-5 inhibitors. However, our retrospective analysis gives some hints, which could be useful for the design of future prospective studies in cardiovascular prevention in diabetic patients and for the identification of the best predictive tool in this setting.

The strength of the present study relies on the study design of the original trial (prospective, double blind, placebo controlled), which provided the longest treatment duration with a PDE-5 inhibitor and follow-up phase published so far. Admittedly, however, the treatment duration is at the same time a potential limit for the parameter investigated here. Indeed, cardiovascular risk is predicted at ten years and longer treatment durations might be necessary to influence this parameter. Finally, the low number of patients represents another important limit.

In conclusion, despite six-months of chronic Vardenafil administration do not change cardiovascular risk prediction, a beneficial effect of PDE-5 inhibitors on cardiovascular health, via the previously demonstrated action on inflammatory markers is suggested. Similarly, the PDE-5 inhibitors effect in restoring normal gonadal function seems to influence the prediction of long term cardiovascular risk. Longer duration of chronic PDE-5 inhibitors treatment should be evaluated in the near future.

## Supporting information

S1 File CONSORT 2010 checklist. (DOC)

S2 File Original study protocol. (PDF)

S3 File Complete dataset used for analyses. (PDF)
