# Supplementary material for: Could chronic Vardenafil administration influence the cardiovascular risk in men with type 2 diabetes mellitus?
Source: PLoS One. 2018 Jun 28;13(6):e0199299. doi: 10.1371/journal.pone.0199299 (PMC6023114; doi:10.1371/journal.pone.0199299)
Supplement: S2 File — (PDF) [file pone.0199299.s002.pdf]

Study Protocol

Monitoring of endothelial dysfunction during chronic administration of vardenafil in patients with type 2 diabetes mellitus: A longitudinal, randomised, placebo-controlled, double blind, phase II b, clinical trial

EudraCT number 2009-014137-25

Principal Investigator

Prof. Cesare Carani

Other Investigators:

Prof. Manuela Simoni, Dr. Antonio Granata and Dr. Augusto Baldini

Institution:

Integrated Department of Medicine, Endocrinology, Metabolism and Geriatrics,
University of Modena and Reggio Emilia, Italy

Address:
Nuovo Ospedale Civile S. Agostino Estense
Via Giardini 1355
I – 41100 Modena, Italy
Tel: 0039-396 1815
Fax: 0039-396 1335
e-mail: cesare.carani@unimore.it

Content

page

1. Summary……………………………………………………………………………3
2. Background and rationale of the study…………………………………….......4
3. Objectives of the study………………………………………………………...13
4. Design and methodology……………………………………………………...13
5. Expected outcome……………………………………………………………...33
6. Main problems anticipated…………………………………………………....34
7. Adverse events…………………………………………………………………34
8. References……………………………………………………………………...37
9. The study at a glance (synoptic table)………………………………………….42

1. Summary

Phosphodiesterase-5 (PDE5) inhibitors are currently used in the therapy of erectile dysfunction (ED), which is considered to be an early symptom of cardiovascular disease (CD). Impairment of endothelial function is the early lesion preceding atherosclerosis and is common to ED, CD and vascular complications of diabetes mellitus (DM). Continuous administration of the PDE5 inhibitors tadalafil or sildenafil for four weeks in men with increased cardiovascular risk or type 2 DM has been shown to improve endothelial function measured by flow-mediated dilation and reduction of inflammatory markers. The goal of this study is to investigate whether chronic, long-term treatment with vardenafil improves and/or prevents deterioration of systemic endothelial function in patients with type 2 DM. Study design: longitudinal, double-blind, randomised, placebo-controlled interventional study. Patients: 106 male patients (n determined by power analysis) with type 2 DM diagnosed and classified according to the guidelines of the European Association for the Study of Diabetes. Beside the antidiabetic treatment, patients will be randomly assigned to receive either placebo or vardenafil, 10 mg p.o. two times a day for 24 weeks. Exclusion criteria: all contraindications to treatment with PD5 inhibitors. Age range: 40-60 years. Sample size determined by power analysis assuming variations of the primary end point of ± 10% from baseline at 0.05 α level with a power of 80%, a two-sided test and a dropout rate of 15%. Primary end point: serum endothelin 1 levels (decresed/not increased in the verum group vs. not decrease/increased in the placebo group). Other parameters and measurements: Complete clinical workup, weight, height, WH ratio, serum lipids and glucose, HbA1c, micro- and macroalbuminuria, homocysteine, hsCRP, IL-6, PAI, TNFα, fibrinogen, adiponectin, testosterone, SHBG and estradiol. All parameters measured at baseline, 4 weeks, 12 weeks and 24 weeks on medication as well as 4 and 24 weeks after discontinuation of the study drug. Study duration: two years. Statistics: descriptive statistics, ANCOVA and multiple regression analysis or non-parametric tests as appropriate by STATA. Ethics: The trial will be registered. Ethics committee approval and patient informed consent will be obtained. Expected outcome and objective: Monitoring of endothelial function deterioration in type 2 DM during chronic administration of Vardenafil. Possibility for a long term-follow up study. Publication in a high impact factor journal.

2. Background and rationale of the study

2.1 Diabetes mellitus: epidemiology and socioeconomic impact

The prevalence of diabetes is rapidly increasing worldwide due to population growth, ageing, urbanisation and increasing prevalence of obesity and physical inactivity. A recent study estimated that the prevalence of diabetes for all age-groups worldwide was 2.8% in 2000 and will rise to 4.4% in 2030. The total number of people with diabetes is projected to rise from 171 million in 2000 to 366 million in 2030 (Wild et al. 2000). The human and economic costs of this epidemic are enormous. The increase of diabetes prevalence especially in developing countries will inevitably result in increasing proportions of deaths from cardiovascular disease and associated consequences as well as other complications of diabetes, requiring innovative preventive and therapeutic approaches.

In Italy the prevalence of type 1 diabetes mellitus is estimated between 0.4 and 1 pro thousand, while type 2 diabetes ranges between 3 and 11 %. The economic impact of diabetes in Italy is estimated to be around 5.17 millions EURO per year, accounting alone for 6.65 % of the total public expenditures for health. The costs for the national health system increase three-four folds in the presence of complications related to micro- or macroangiopathy, five folds in the presence of both. Therefore, therapeutic strategies involving prevention (including secondary prevention of complications) not only improve quality of life of the patients but will have a dramatic economic impact (Ministero della Salute 2000).

2.2 Endothelial dysfunction in diabetes mellitus

Diabetes mellitus is associated with both macro- and microvascular angiopathy, resulting in atherosclerosis with increased prevalence of cardiovascular disease and retinopathy/ nephropathy, respectively. The pathogenetic mechanism of vascular complications of diabetes is not fully understood but many recent evidences focus on the crucial role of the endothelium (Cosentino and Lüscher 1998; Creager et al. 2003). In essence, it appears that vasodilation mediated by endothelium-derived nitric oxide (NO) is impaired in animal models of diabetes and in patients with type 1 and type 2 diabetes mellitus.

The role of NO as a principal factor involved in the antiatherosclerotic properties of the endothelium is well established. NO is constitutively produced by endothelial NO synthase (NOS) through a 5-electron oxidation of the guanidine-nitrogen terminal of L-arginine and causes vasodilation by activating guanylyl cyclase of the underlying vascular smooth muscle cells (Moncada and Higgs 1993) (Fig. 1). NO has potent vasodilator (Creager et al. 1990), anti-inflammatory (Kataoka et al. 2002), antiproliferative (Tanner et al. 2000), antioxidant (Clapp et al. 2004) and antiplatelet effects (Schäfer et al. 2004). A reduction in NO bioavailability is present in atherosclerotic vessels before

vascular structural changes occur. In fact, longitudinal studies have shown that impaired NO-dependent vasodilation is a predictor of future cardiac events (Schachinger et al. 2000) and of development of coronary artery atherosclerosis (Bugiardini et al. 2004). Reduction of endothelium-derived NO results in activation of the transcription factor Nuclear Factor Kappa B (NF-κB) with pro-inflammatory consequences: increased production of leukocyte adhesion molecules, chemokines and cytokines. These factors promote monocyte and vascular smooth muscle cell migration into the intima and formation of macrophage foam cells, i.e. the initial morphological changes of atherosclerosis (Zhang 2008; Lubos et al. 2008).

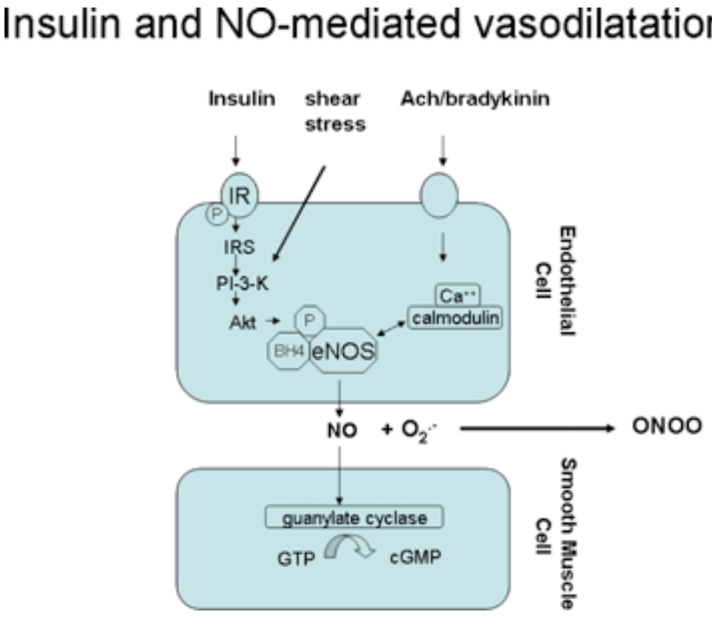

Figure 1. Insulin and endothelial cell NO release Abbreviations: IR, insulin receptor; IRS, insulin receptor substrate; PI3K, phosphatidyl inositol 3-kinase; BH4, tetrahydrobiopterin; eNOS, endothelial nitric oxide synthase; Akt, protein kinase B; P, phosphorylation; and ONOO, peroxynitrite. (from: Kearney et al. 2008)

Endothelial dysfunction, as represented by impaired endothelium-dependent, NO-mediated relaxation, occurs in cellular and experimental models of diabetes and several clinical studies have found that endothelium-dependent vasodilation is abnormal in patients with type 1 or type 2 diabetes (Kearney et al. 2008). Decreased levels of NO may be the most important pathogenetic event in atherogenic predisposition in diabetes. Although the mechanism by which diabetes results in decreased NO availability is not clear, insulin receptor are present on endothelial cells and insulin has been shown to contribute directly to NO production (Fig.1) (Kearney et al. 2008). Bioavailability of NO, however, is the result of a balance between its production via NOS and its degradation, particularly by oxygen-derived free radicals. Some hallmarks of diabetes such as *hyperglycemia*, excess *free fatty acid* liberation, and *insulin resistance*, can affect synthesis and/or degradation of NO and are established causes of endothelial cell dysfunction. (Figure 2).

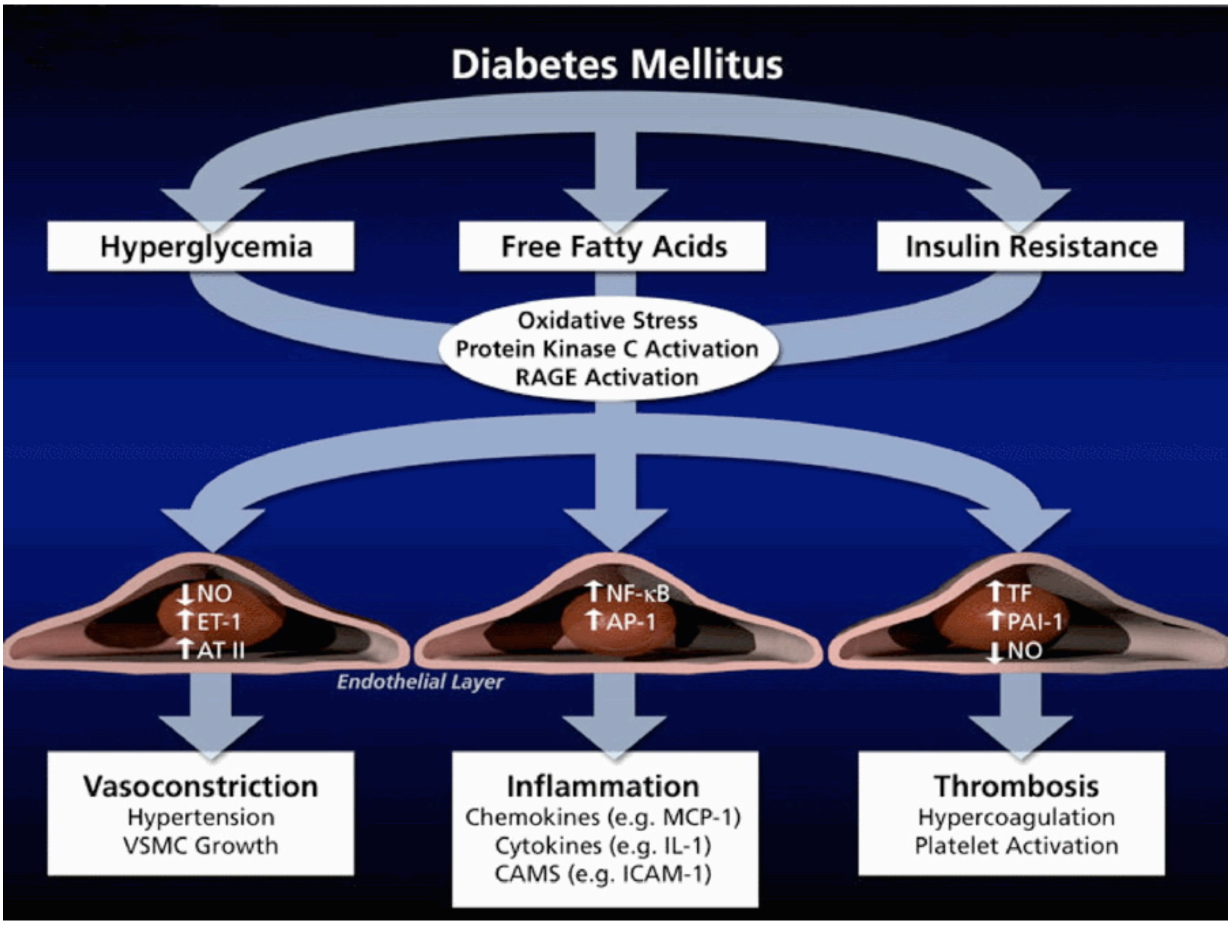

Figure 2. The metabolic abnormalities that characterize diabetes, particularly hyperglycemia, free fatty acids, and insulin resistance, provoke molecular mechanisms that alter the function and structure of blood vessels. These include increased oxidative stress, disturbances of intracellular signal transduction (such as activation of PKC), and activation of RAGE. Consequently, there is decreased availability of NO, increased production of endothelin 1 (ET-1), activation of transcription factors such as NF-κB and AP-1, and increased production of prothrombotic factors such as tissue factor (TF) and plasminogen activator inhibitor-1 (PAI-1). (From Creager et al. 2003)

Hyperglycemia decreases endothelium-derived NO and increases reactive oxygen species by stimulating superoxide anion production via the mitochondrial electron transport chain. This, in turn, promotes a cascade of events (involving activation of PKC and ensuing consequences) resulting in ever-increasing production of superoxide anion and inactivation of NO. Superoxide anion also increases intracellular production of advanced glycation end products (AGEs). These glycated proteins adversely affect cellular function both by affecting protein function and by activation of the receptor for AGEs (RAGE) and further supporting production of oxygen-derived free radicals. Hyperglycemia-induced oxidative stress also may increase levels of asymmetric dimethylarginine, a competitive antagonist of NOS. The concept that hyperglycemia-induced oxidative stress mediates endothelial dysfunction in patients with diabetes is supported by the observations that intra-arterial infusion of ascorbic acid, a water-soluble antioxidant capable of

scavenging superoxide anion, restores endothelium-dependent vasodilation in patients with type 1 or type 2 diabetes (Ting et al. 1996; Timini et al. 1998).

Free fatty acids may impair endothelial function through several mechanisms, including increased production of oxygen-derived free radicals, and exacerbation of dyslipidemia. Since co-infusion of the antioxidant ascorbic acid improves endothelium-dependent vasodilation in humans treated with free fatty acids, oxidative stress should be involved. Elevation of free fatty acid concentrations activates PKC and decreases NOS activity. In addition, hypertriglyceridemia and low HDL, resulting from increased free fatty acid availability to the liver, have been associated with endothelial dysfunction (Creager et al. 2003).

Type 2 diabetes mellitus is characterized by *insulin resistance*. Insulin stimulates NO production directly in endothelial cells (Fig. 1) by increasing the activity of NOS via activation of phosphatidylinositol-3 kinase (PIK3) and Akt kinase. Thus, in healthy subjects, insulin increases endothelium-dependent (NO-mediated) vasodilation. In insulin-resistant subjects, endothelium-dependent vasodilation is reduced. Drug therapies that increase insulin sensitivity, such as metformin and the thiazolidinediones, improve endothelium-dependent vasodilation (Mather et al. 2001). Abnormal endothelium-dependent vasodilation in insulin-resistant states depends on reduced activation of the PIK3 pathway so that insulin is less able to activate NOS and produce NO. On the contrary, insulin signaling via the mitogen-activated protein kinase pathway remains intact and is associated with increased endothelin 1 production, promoting inflammation and thrombosis (Ferri et al. 1995).

Following oxidative stress and reduced NO bioavailability, the endothelium reacts with increased production of factors promoting vasoconstriction, inflammation and thrombosis (Fig. 2). In particular endothelin 1 promotes inflammation and causes vascular smooth muscle cell contraction and growth. Insulin increases endothelin 1 immunoreactivity in endothelial cells and plasma endothelin 1 concentration increases after administration of insulin to healthy subjects and patients with type 2 diabetes mellitus (Ferri et al. 1995; Piatti et al. 1996). Other consequences of oxidative stress and PKC activation are dysregulation of vascular smooth muscle cell function, with migration into the atherosclerotic lesion, proliferation and production of extracellular matrix, and abnormal platelet function, hypercoagulation and increased production of plasminogen activator inhibitor 1 (PAI-1), with reduced fibrinolysis (Creager et al. 2003).

In summary, the vascular disease observed in diabetes, which involves both the micro- and macrovasculature, derives from a reduced bioavailability of endothelium-derived NO, which could be regarded as the *primum movens* of the vasculopathy. Yet, the pharmacological strategies to

improve systemic endothelial function in diabetes have been so far indirect at best, being based on targeting hyperglycemia and insulin resistance, hypertension, dyslipidemia and hypercoagulation. Strict glycemic control may improve microangiopathy (The ADVANCE 2008) but is unable to reduce cardiovascular events (The ACCORD 2008; Duckworth et al. 2009), increasing on the contrary body weight and the risk of hypoglycemia. Insulin sensitizers (e.g. thiazolidinediones), angiotensin-converting enzyme inhibitors, angiotensin II receptor blockers, statins and aspirin have indirect beneficial effects on endothelial function but do not act on the *primum movens*. An approach based on increasing the availability of cGMP, i.e. the effector of NO-mediated vasodilation (e.g. by PDE5 inhibitors) to stably improve systemic endothelial function in diabetes mellitus was not attempted so far.

2.3 PDE5 inhibitors and endothelial function

2.3.1 Types of phosphodiesterases (PDE) and function of PDE5

Cyclic nucleotide phosphodiesterases (PDEs) are enzymes that regulate the cellular levels of the second messengers, cAMP and cGMP, by controlling their rates of degradation. There are 11 different PDE families, with each family having several different isoforms and splice variants (reviewed by Bender and Beavo 2006). These unique PDEs differ in their three-dimensional structure, kinetic properties, modes of regulation, intracellular localization, cellular expression, and inhibitor sensitivities. Current data suggest that individual isozymes modulate distinct regulatory pathways in the cell, offering the opportunity for selectively targeting specific PDEs for treatment of specific disease.

PDE5 was originally identified, isolated, and characterized from platelets and lung but received little attention until it was discovered to be a regulator of vascular smooth muscle contraction and more importantly the target for the drug sildenafil. PDE5 is characterized by a relative specificity for cGMP hydrolysis at low substrate levels and by the presence of high affinity-binding sites for cGMP. Only one PDE5 gene has been discovered to date, *PDE5A*, although several variants under the control of differentially regulated promoters have been identified.

PDE5A is a cytosolic protein. In rodents, relatively high levels of *PDE5A* mRNA have been localized to vascular smooth muscle, heart, placenta, skeletal muscle, pancreas, brain, liver, several gastrointestinal tissues and lung. The highest levels of *PDE5A* mRNA are found in the cerebellum, kidney, and pancreas and prominent expression is also seen in lung and heart. PDE5A protein is also expressed in platelets and is prominently expressed in smooth muscle including the vascular tissues of the penis. There are different *PDE5A* expression variants denominated *PDE5A1*, *PDE5A2* and *PDE5A3.* The expression of the latter seems to be restricted to vascular smooth muscle (Lin et al. 2002).

In the vascular smooth muscle PDE5 is as a regulator of contraction. Two tissues in which this role is evident are the lung and the penis. In the cavernosal smooth muscle of the penis, PDE5 inhibition enhances relaxation of smooth muscle by NO and cGMP and thereby stimulates penile erection (Rosen and Kostis 2003; Corbin 2004). In the lung, inhibition of PDE5 opposes smooth muscle vasoconstriction, and PDE5 inhibitors are now used for treatment of pulmonary hypertension. Similarly, PDE5 is also thought to be important in the regulation of platelet aggregation. Inhibition of PDE5 in the presence of NO increases platelet cGMP and enhances the inhibitory effect of NO on platelet aggregation and secretory function (Ito et al. 1996; Dunkern and Hatzelmann 2005). PDE5 has also been implicated to play a role in learning and memory (Prickaerts et al. 2004) and in cardiac remodelling, since sildenafil prevents pressure-induced cardiac hypertrophy in several models of cardiac hypertrophy and heart failure due to pressure overload (Takimoto et al. 2005).

2.3.2 PDE5 inhibitors

The PDE5 inhibitors currently commercially available are sildenafil (Viagra), tadalafil (Cialis), and vardenafil (Levitra). These drugs have proven very effective for the treatment of erectile dysfunction and are being tested for other pathological conditions as well.

The half-life and duration of action of the three drugs differs. While sildenafil and vardenafil have a half life of about 4 hours and 4.8 hours respectively and have a duration of action of about 4 hours, tadalafil has a half life of about 17.5 hrs and its action lasts for about 36 hours. The overall safety of PDE5 inhibitors is good. There is a risk of hypotension if nitrates are given concurrently so that the drugs should not be given to patients assuming nitrates. Common side-effects include headache, facial flushing, nasal congestion, dyspepsia and transient visual impairment and are reported by 3% of patients. These effects suggest additional roles for cGMP (and PDE5) in these tissues. To date, however, the molecular mechanisms for these side effects are not well understood. There are pharmacological interactions between these drugs and other medications metabolized by the cytochrome P450 (P3A4 isoform), such as the azole antifungals, erythromycin and the HIV protease inhibitors.

2.3.3 PDE5 inhibitors for the treatment of erectile dysfunction (ED)

Erectile dysfunction (ED) is defined as the inability to achieve and maintain an erection sufficient to permit satisfactory sexual intercourse (NIH Consensus 1993) is estimated to afflict over 100 million men worldwide. In most cases ED recognises a vascular ethiology and is currently very effectively treated with PDE5 inhibitors (Setter et al. 2005). As indicated above, this class of drugs augments NO effects on the arterial wall inducing smooth muscle relaxation and arterial dilation in several

body districts, including the *corpora cavernosa*, an effect dependent on availability of cGMP. By preventing breakdown of cGMP PDE5 inhibitors enhance penile erection. When sexual stimulation releases NO into the penile smooth muscle, inhibition of PDE5 causes a marked elevation of cGMP concentrations in the *glans penis*, *corpus cavernosum* and *corpus spongiosum*, resulting in better erection. PDE5 inhibitors have no effect on the penis in the absence of sexual stimulation, when the concentrations of NO and cGMP are low (Lue 2000).

The efficacy of PDE5 inhibitors on ED on one hand demonstrates the vascular origin of this condition in most cases, on the other hand reveals ED as an early symptom of subclinical atherosclerotic cardiovascular disease. In fact, patients with ED have significantly higher levels of inflammatory markers (hs-CRP) and coronary artery calcifications and significantly impaired flow-mediated dilation (FMD) of the brachial artery compared to men without ED (Chiurlia et al. 2005). Very often atherosclerotic cardiovascular disease and ED are linked also at the clinical level: ED is common in patients with overt and silent coronary artery disease. Therefore ED is increasingly being regarded as the early clinical manifestation of a generalized vascular disease and is an independent risk for future cardiovascular events.

Chronic treatment of erectile dysfunction with PDE5 inhibitors was attempted in order to ascertain whether long term administration of the drug would result in stable “rehabilitation” of the endothelium with maintenance of erectile function also after dismissing the medication. In a prospective, open-label trial patients with ED treated with sildenafil 50 mg at bedtime over a 1-year period demonstrated to retain normal erectile function and improved arterial blood flow during pharmacologically induced erection even six months after drug discontinuation (Sommer and Schulze 2005; Sommer et al. 2007). Positive effects of chronic administration over a shorter period (four weeks) were seen with tadalafil as well. In men with ED not only sexual function improved and was maintained for two weeks after discontinuation but also inflammation markers such as endothelin 1, VCAM and CRP decreased significantly with this regimen with a concomitant increase in serum insulin levels (Aversa et al. 2007).

2.3.4 PDE5 inhibitors in the treatment of ED in diabetic men

Over 50% of diabetic men develop ED within 10 years of diagnosis of diabetes (Richardson and Vinik 2002). ED in diabetes results from a combination of impairments affecting nearly every step in the production of a penile erection. These include the failed transmission of neural signals to and from the spinal cord due to neuropathy resulting in reduced neural NO delivery to cavernosal smooth muscle, impaired sinusoidal endothelial cell NO release because of endothelial dysfunction, reduced arterial and arteriolar inflow due to peripheral vascular disease, and failure of

relaxation of the *corpora cavernosa* from glycation of the elastic fibres. PDE5 inhibitors are very helpful in the treatment of ED in diabetic men, a group in which this sexual condition is very difficult to treat (Basu and Ryder 2004).

All three PDE5 inhibitors taken *as needed* demonstrated to be very effective in improving ED and are well tolerated in patients with diabetes studied over relatively long time periods of several weeks (Vardi and Nini 2007). Recently, *continuous* treatment with PDE5 inhibitors has been proposed, addressing ED management as any other chronic condition. A randomized, double-blind, placebo-controlled, multicentre, 12-week study based on 298 men with diabetes and ED who received once-daily treatment with placebo, tadalafil 2.5 mg or tadalafil 5 mg, showed that patients receiving tadalafil had significant improvements of erectile function and the drug was well tolerated (Hatzichristou et al. 2008). The serum concentrations of the inflammatory and metabolic parameters CRP, nitrotyrosine, ICAM-1 or VCAM-1, HbA1c, fructosamine and the urinary albumin/creatinine ratio (ACR) did not significantly change from baseline to end-point but the study suffered from imperfect randomisation. Indeed in a *post hoc* analysis, patients who received tadalafil 2.5 and 5 mg and who were in the highest baseline tertile of ACR values (> 32 mg/g) (i.e. those whose ACR suggested incipient diabetic nephropathy) had a significant decrease in ACR and albuminuria over the study period. This suggests a possible benefit of chronic administration of PDE5 inhibitors on the systemic atherosclerotic damage in diabetics, given that microalbuminuria is a significant predictor of asymptomatic coronary artery disease (CAD) in diabetic patients and that ED is an efficient predictor of silent CAD in this population (Gazzaruso et al. 2004). This observation expands the possible spectrum of use of PDE5 inhibitors in the treatment/prevention of diabetic vascular damage well beyond the penile district.

2.3.5 The expanding spectrum of usage of PDE5 inhibitors

Endothelial damage occurs before the atherosclerotic plate can be identified. There are many biomarkers for inflammation and early endothelial dysfunction that can be used to identify endothelial damage in order to prevent cardiovascular disease (CD), including: high sensitivity C reactive protein (hs-CRP), homocysteine, microalbuminuria, glycosylated hemoglobin (A1c), von Willebrand factor and plasminogen-activator inhibitor type 1 (PAI-1). Elevated hs-CRP appears to be a strong predictor for future cardiovascular events Elevated serum homocysteine levels have been associated with a greater risk of developing thrombosis, as well as an increase in the production of hydrogen peroxide and in the oxidation of low-density lipoprotein cholesterol (LDL-C), leading to endothelial damage. Microalbuminuria is an important risk factor for diabetic nephropathy serves as a predictor of CD. In patients with a high risk of developing CD, such as

diabetic patients, it is important to improve endothelial function since vascular damage may continue even when good glycemic control has been achieved.

PDE-5 inhibitors have cardiovascular effects. For instance, they inhibit PDE-5 enzymes in pulmonary vasculature, causing vasodilation and decreasing pulmonary vascular pressure. Indeed sildenafil is approved for treatment of patients with pulmonary hypertension. PDE-5 inhibition with sildenafil improves cardiac output by balancing pulmonary and systemic vasodilation, and augments and prolongs the hemodynamic effects of inhaled nitric oxide in patients with chronic congestive heart failure and pulmonary hypertension (Kapur et al. 2008). Ongoing *in vivo* and *in vitro* studies are examining the possible beneficial effects of PDE-5 inhibitors in conditions such as myocardial infarction and endothelial dysfunction in general. For instance, several small studies and case reports have shown beneficial effects of PDE5 inhibitors on microcirculation with symptomatic relief in patients with Raynaud's phenomenon (Levien 2006).

In experimental models PDE5 inhibitors protect against ischemic phenomena and reduces the possibility of myocardial infarction after inducing ischemia. In diabetic rats long term administration of a PDE5 inhibitor improves endothelial function and decreases plasma endothelin 1 levels (Ahn et al. 2005; Schäfer et al. 2008). These changes are supported by biochemical and histological modifications such as an improved immunohistochemical staining of endothelial and smooth muscle cells and changes in protein expression in diabetic rats receiving vardenafil for 6 weeks (De Young et al. 2008). In the rabbit vardenafil can improve diabetic nephropathy. In alloxan-induced diabetic rabbits vardenafil (3 mg/kg for 4 weeks) significantly reduced serum creatinine and reduced proteinuria, highlighting a potential role for vardenafil in the treatment of diabetic nephropathy (Lau et al. 2007).

Considering that, in diabetic patients, PDE5 inhibitors are used in the management of ED and that endothelial dysfunction underlies both ED and cardiovascular disease in such patients, Desouza et al. (2002) investigated the acute and prolonged effects of sildenafil on flow-mediated dilation (FMD), an accepted method for the non-invasive evaluation of endothelial function. A significant improvement of FMD was observed both acutely and 24 hrs after withdrawal of sildenafil (25 mg) administered daily for 14 days, leading to the hypothesis that prolonged treatment with PDE5 inhibitors may have beneficial effects on vascular function well beyond ameliorating ED in diabetic patients (Desouza et al. 2002).

Another placebo-controlled study investigated the effects of daily administration of sildenafil (50 mg) for 30 days to diabetic patients on microalbuminuria, HbA1c, hsCRP and homocysteinemia. Treatment was demonstrated to effectively and significantly reduce both microalbuminuria and,

interestingly, HbA1c, while no effect were seen on serum homocysteine and hrCRP, possibly due to the short duration of therapy (Grover-Pàez et al. 2007).

In a double-blind, placebo-controlled study, 20 patients with type 2 diabetes mellitus *without* erectile dysfunction randomly received sildenafil or placebo for 4 weeks. In patients allocated to chronic sildenafil, a progressive increase in percentage of patients with FMD improvement was noted while a progressive decrease in the placebo group occurred. In addition, a decrease in endothelin 1 levels and an increase in nitrite/nitrate levels were found after chronic sildenafil; significant changes from baseline in CRP, interleukin 6, intercellular adhesion molecule and vascular adhesion molecule levels were also found (Aversa et al. 2008). This study demonstrated that daily sildenafil administration improves endothelial function and reduces markers of vascular inflammation, suggesting that the impairment of endothelial function resulting in micro- and macroangiopathy in diabetic men may be improved by prolonged PDE5 inhibition.

3. Objectives of the study

Given the protective effect of NO on the endothelium and the results obtained so far in short-term, continuous treatment with PDE5 inhibitors on parameters of endothelial function, we hypothesise that chronic treatment with vardenafil can prevent or delay the deterioration of systemic endothelial function in patients with type 2 diabetes mellitus. The favourable effect of PDE5 inhibitors on sexual function in these patients has been convincingly demonstrated in the past. Here we hypothesise that vardenafil treatment can have beneficial effects on the vascular physiology in other body districts, possibly preventing the development of microangiopathy and atherosclerotic cardiovascular disease in these patients.

The main goal of this study is therefore to monitor the endothelial dysfunction during continuous treatment with vardenafil for 6 months; object of the study will be endothelin 1 and other known parameters of endothelial damage in newly diagnosed patients with type 2 diabetes mellitus.

4. Design and Methodology

4.1 Study design and general outline

This is a longitudinal, randomised, placebo-controlled, double-blind, phase IIb clinical trial involving patients with type 2 diabetes mellitus followed up at the Antidiabetic Centre (tertiary care centre) of the Integrated Department of Medicine, Endocrinology, Metabolism and Geriatrics of the University of Modena and Reggio Emilia. The study protocol consists of a Screening/Enrolment Phase lasting up to 4 weeks, a Treatment Phase of 24 weeks, and Follow-up/Observation Treatment-free Phase

of 24 weeks after the Treatment Phase. Patients with type 2 diabetes mellitus diagnosed within the last 12 months according to the guidelines of the European Association for the Study of Diabetes (2007) and treated as appropriate will be asked to participate to the study.

After informed consent is provided, the patients will enter a Screening/Enrolment Phase consisting of two visits as indicated below (3.6). Enrolled patients will then randomly allocated to receive, beside the antidiabetic treatment, placebo or vardenafil, 10 mg p.o. twice a day for 26 weeks. The study medication will then be dismissed and patients will enter a observation phase of 24 weeks. The complete duration of the study will be two years.

4.2 End points and calculation of sample size

The primary end point is the serum endothelin 1 concentration (decreased/not increased in the verum group vs. not decrease/increased in the placebo group). The sample size has been determined by power analysis assuming variations of the primary end point (serum endothelin 1) of ± 10% from baseline, repeated measurements after baseline (ANCOVA), at 0.05 α level with a power of 80%, a two-sided test and a dropout rate of 15%. A clinically meaningful difference (delta) of 0.35 pg/ml with a SD of 0.8 pg/ml in the verum group has been assumed. Power analysis (by STATA 8.2) indicated a sample size of 53 patients per arm. The 10% variation compared to baseline is a conservative hypothesis based on the results of a previous study of shorter duration (Rosano et al., 2005).

4.3 Subjects and selection criteria

106 male patients affected by type 2 Diabetes mellitus will be involved in this study. Diabetes mellitus should be diagnosed within 12 months before enrolment in the study, according to the guidelines of the European Association for the Study of Diabetes (2007). Only subjects affected by mild or severe ED will be enrolled in the study.

The patients will be under the most appropriate antidiabetic treatment, which should be continued for the duration of the study. Depending on the individual patient, this treatment may include both oral drugs for glycemic control and/or insulin, as appropriate. Other drugs commonly used in diabetic patients (e.g. statins, anticoagulants, blood pressure regulators, etc..) may be used as indicated. The type of treatment received by each patient enrolled will be noted on the CRF.

4.3.1 Inclusion criteria

- Male gender;
- Signed written Consent Form
- Type 2 diabetes mellitus diagnosed within 5 years before enrolment in the study;

- Body Mass Index (BMI) < 35;
- ED (severe, moderate or light according to the International Index of Erectile Function – erectile function domain score < 26 (Rosen et al. 1997));
- Age range: 40-65 years.

4.3.2 Exclusion criteria

- Systemic diseases except type 2 diabetes mellitus;
- Absence of ED;
- Psychiatric disorders;
- All contraindications to treatment with PDE5 inhibitors:

    retinitis pigmentosa, non-arteritic anterior ischaemic optic neuropathy, history of coronary artery disease, recent myocardial infarction, unstable angina, systemic arterial hypotension, nitric-oxide donors therapy, severe hepatic and renal failure.

4.4 Subjects allocation/randomization

All volunteers who sign a Consent Form and meet the entry and screening criteria will be assigned a subject screening number, and go through the Screening/Enrolment Phase of the study.

Randomisation will be performed by the permuted block method (Beller et al. 2002).

4.5 Study medication

Tablets of vardenafil (10 mg) and placebo will be prepared and provided by Bayer Schering Pharma, Berlin, Germany.

Vardenafil pharmacokinetics (data provided by Bayer-Schering) show the following characteristics: a) maximum plasma concentrations after a single 20 mg dose usually reached between 30 minutes and 2 hours after oral dosing in the fasted state; b) the terminal half-life of vardenafil and its primary metabolite is approximately 4-5 hours; c) high-fat meals cause a reduction in maximum concentration (C max) by 18%-50%; d) the recommended dose is between 5 and 20 mg once per day; the maximum overdosage data in humans concern a single 120 mg dose administered to healthy men, the majority of whom experienced reversible back pain, myalgia and/or vision disturbances.

Vardenafil adverse reactions (data provided by Bayer-Schering) are headache (15%), flushing (11%), rhinitis (9%), dyspepsia (4%), sinusitis and flu-syndrome (3%), dizziness and nausea (2%).

Absolute contraindications to vardenafil administration are retinitis pigmentosa, non-arteritic anterior ischaemic optic neuropathy, recent myocardial infarction, unstable angina, systemic arterial hypotension, nitric-oxide donors therapy, severe hepatic and renal failure.

Caution is advised when vardenafil is co-administered with alpha-adrenergic blocking agents, because of possible additive effect on blood-pressure due to combined assumption of vasodilators and leading to symptomatic hypotension. On the base of vardenafil pharmacokinetics and with the aim to achieve and maintain effective plasma level as constant as possible with the least number of administrations/day, the enrolled subjects will receive placebo or vardenafil, 10 mg p.o., twice per day (b.i.d.). Patients will be instructed to assume tablets one hour before lunch and one hour before bedtime, in any case at least one hour before or at least three hours after food intake.

4.6 Screening and enrolment procedure

During this phase (lasting 2-4 weeks), volunteers will undergo 2 clinic visits (visit 1 and visit 2) before enrolment. The initial visits include:

VISIT 1:

Anamnesis and verbal screening for eligibility criteria. The patient will be informed about the aims, purposes and possible risks of the study and provided with an information sheet. If eligible, willing and interested in participating in the study the patient will undergo complete clinical workup including blood pressure, cardiac frequency and electrocardiogram, weight, height and waist/hip ratio and andrological examination, the latter consisting in objective clinical evaluation of both volume and consistency of breast, testes and penis. Both an urine and a blood (8 ml) sample will be taken for assays routinely performed in the follow up of diabetes therapy.

The following biochemical parameters will be measured at visit 1

Routine blood assay:

- Hemochrome (including White Cells)
- Glucose
- Glycated hemoglobin (HbA1c)
- Creatinine
- Urea
- Glutamic-oxalacetic transaminase (GOT)
- Glutamic-pyruvic transaminase (GPT)
- Alcaline phosphatase
- Triglicerids
- Total cholesterol (t-CH)

Low-Density Lipoproteins (LDL-CH)
High-Density Lipoproteins (HDL-CH)
Apo-protein AI (Apo-AI)
Apo-protein B (Apo-B)
Apo-protein C-III (Apo-CIII)
Eritrosedimentation speed (ES)
High sensitivity C-Reactive protein (hsCRP)
Endothelin 1 (ET-1)

Urine assay:
Microalbuminuria
Macroalbuminuria
Physical and chemical urine examination

In addition the patient will undergo an unstructured sexological interview and provided with questionnaires to be filled during the visit: the self-filled questionnaire "International Index of Erectile Function" (Rosen et al. 1997) and the self-filled questionnaire "Beck's Depression Inventory" (Beck et al. 1961). All data will be recorded on the Case Report Form (CRF) provided.

4.7 Treatment phase

4.7.1 Second screening visit and start point of treatment phase (24 weeks)

VISIT 2 (within 4 weeks of visit 1; week 1 of treatment phase):
Second screening visit and start point of treatment phase.
The investigator will review the results of analyses and screening tests from Visit 1 with the patient. If eligible, the patient will sign the informed consent and will be enrolled in the study. The patient will be randomised and will receive the participant identification number which will be entered in the identification section of the CRF. The randomisation envelope will be kept together with the patient's study documentation. Once a participant identification number has been assigned it cannot be reassigned to another participant, even if the participant receives no study treatment for any reason. Patients screened but not eligible or not willing to participate will be recorded on separate file. Additional participants recruited to the study to achieve the desired number completing the treatment schedule are assigned consecutive participant numbers in the order that they are identified as eligible.

If a participant identification number is assigned, this visit is considered the first visit of the Treatment Phase. Baseline biochemical measurements will not be repeated at this visit but two further blood samples (8 ml in total) will be taken for storage of plasma and serum

(–20°C) for the measurement of sexual hormones and of parameters of endothelial function to be performed at the end of the study.

Parameters of endothelial function:

- Interleukin 6 (IL-6)
- Tumor necrosis factor alfa (TNF-alfa)
- Von Willebrand factor (vWF)
- Fibrinogen
- Plasminogen activator 1 (PAI-1)
- Homocysteine
- Adiponectin

Sexual hormones:

- Luteinizing-hormone (LH)
- Follicular-stimulating- hormone (FSH)
- Testosterone
- Estradiol (E2)
- Sex-hormone binding globulin (SHBG)

Another blood sample for genomic DNA will be collected and stored for post-hoc analysis of genetic determinants of response.

The following instrumental investigations will be performed: Flow-mediated dilation (FMD) and pulse wave velocity (PWV)

Study medication will be handed out to the patient, who will be instructed about the mode and time of administration. A diary form to report possible side effect or any other information connected with the drug assumption will be provided. The patients will be requested to return the diary form and the medication boxes/blister at the next visit. During the treatment phase the patients should return every four weeks (visits 3-8).

4.7.2 Follow-up procedures (visits 3-8)

VISIT 3 (week 2 of the treatment phase):
The patient will return the diary form and the empty medication boxes/blister. New diary forms and medication boxes/blister will be handed out. The investigator will interview the patient and the following clinical parameters will be taken and recorded:

Blood pressure, cardiac frequency and electrocardiogram.

Both an urine and a blood (8 ml) sample will be taken for assays routinely performed in the follow up of diabetes therapy:

Routine blood assay:

- Hemochrome (including White Cells)
- Glucose
- Glycated hemoglobin (HbA1c)
- Creatinine
- Urea
- Glutamic-oxalacetic transaminase (GOT)
- Glutamic-pyruvic transaminase (GPT)
- Alcaline phosphatase
- Triglicerids
- Total cholesterol (t-CH)
- Low-Density Lipoproteins (LDL-CH)
- High-Density Lipoproteins (HDL-CH)
- Apo-protein AI (Apo-AI)
- Apo-protein B (Apo-B)
- Apo-protein C-III (Apo-CIII)
- Eritrosedimentation speed (ES)
- hsCRP
- Endothelin 1 (ET-1)

Urine assay:

- Microalbuminuria
- Macroalbuminuria
- Physical and chemical urine examination

Two further blood samples (8 ml in total) will be taken for storage of plasma and serum (–20°C) for the measurement of parameters of endothelial function to be performed at the end of the study:

Parameters of endothelial function:

- Interleukin 6 (IL-6)
- Tumor necrosis factor alfa (TNF-alfa)
- Von Willebrand factor (vWF)

Fibrinogen
Plasminogen activator 1 (PAI-1)
Homocysteine
Adiponectin

VISIT 4 (week 4 of the treatment phase):
The patient will return the diary form and the empty medication boxes/blister. New diary forms and medication boxes/blister will be handed out. The investigator will interview the patient and the following clinical parameters will be taken and recorded:

Blood pressure, cardiac frequency and electrocardiogram, weight and waist/hip ratio.

Both an urine and a blood (8 ml) sample will be taken for assays routinely performed in the follow up of diabetes therapy:

Routine blood assay:
Hemochrome (including White Cells)
Glucose
Glycated hemoglobin (HbA1c)
Creatinine
Urea
Glutamic-oxalacetic transaminase (GOT)
Glutamic-pyruvic transaminase (GPT)
Alcaline phosphatase
Triglicerids
Total cholesterol (t-CH)
Low-Density Lipoproteins (LDL-CH)
High-Density Lipoproteins (HDL-CH)
Apo-protein AI (Apo-AI)
Apo-protein B (Apo-B)
Apo-protein C-III (Apo-CIII)
Eritrosedimentation speed (ES)
hsCRP
Endothelin 1 (ET-1)
Urine assay:
Microalbuminuria

Macroalbuminuria
Physical and chemical urine examination

Two further blood samples (8 ml in total) will be taken for storage of plasma and serum (–20°C) for the measurement of parameters of endothelial function to be performed at the end of the study:

Parameters of endothelial function:
Interleukin 6 (IL-6)
Tumor necrosis factor alfa (TNF-alfa)
Von Willebrand factor (vWF)
Fibrinogen
Plasminogen activator 1 (PAI-1)
Homocysteine
Adiponectin

VISIT 5 (week 8 of the treatment phase):
The patient will return the diary form and the empty medication boxes/blister. New diary forms and medication boxes/blister will be handed out. The investigator will interview the patient and the following clinical parameters will be taken and recorded:

Blood pressure, cardiac frequency and electrocardiogram.

Both an urine and a blood (8 ml) sample will be taken for assays routinely performed in the follow up of diabetes therapy:

Routine blood assay:
Hemochrome (including White Cells)
Glucose
Glycated hemoglobin (HbA1c)
Creatinine
Urea
Glutamic-oxalacetic transaminase (GOT)
Glutamic-pyruvic transaminase (GPT)
Alcaline phosphatase
Triglicerids
Total cholesterol (t-CH)

Low-Density Lipoproteins (LDL-CH)
High-Density Lipoproteins (HDL-CH)
Apo-protein AI (Apo-AI)
Apo-protein B (Apo-B)
Apo-protein C-III (Apo-CIII)
Eritrosedimentation speed (ES)
hsCRP
Endothelin 1 (ET-1)

Urine assay:

Microalbuminuria
Macroalbuminuria
Physical and chemical urine examination

In addition the patient will undergo an unstructured sexological interview and provided with questionnaires to be filled during the visit: the self-filled questionnaire “International Index of Erectile Function” (Rosen et al. 1997) and the self-filled questionnaire “Beck’s Depression Inventory” (Beck et al. 1961).

VISIT 6 (week 12 of the treatment phase):
The patient will return the diary form and the empty medication boxes/blister. New diary forms and medication boxes/blister will be handed out. The investigator will interview the patient and the following clinical parameters will be taken and recorded:

Blood pressure, cardiac frequency and electrocardiogram, weight and waist/hip ratio.

Both an urine and a blood (8 ml) sample will be taken for assays routinely performed in the follow up of diabetes therapy:

Routine blood assay:

Hemochrome (including White Cells)
Glucose
Glycated hemoglobin (HbA1c)
Creatinine
Urea
Glutamic-oxalacetic transaminase (GOT)
Glutamic-pyruvic transaminase (GPT)
Alcaline phosphatase

Triglicerids

Total cholesterol (t-CH)

Low-Density Lipoproteins (LDL-CH)

High-Density Lipoproteins (HDL-CH)

Apo-protein AI (Apo-AI)

Apo-protein B (Apo-B)

Apo-protein C-III (Apo-CIII)

Eritrosedimentation speed (ES)

hsCRP

Endothelin 1 (ET-1)

Urine assay:

Microalbuminuria

Macroalbuminuria

Physical and chemical urine examination

Two further blood samples (8 ml in total) will be taken for storage of plasma and serum (–20°C) for the measurement of parameters of endothelial function to be performed at the end of the study:

Parameters of endothelial function:

Interleukin 6 (IL-6)

Tumor necrosis factor alfa (TNF-alfa)

Von Willebrand factor (vWF)

Fibrinogen

Plasminogen activator 1 (PAI-1)

Homocysteine

Adiponectin

VISIT 7 (week 18 of the treatment phase):

The patient will return the diary form and the empty medication boxes/blister. New diary forms and medication boxes/blister will be handed out. The investigator will interview the patient and the following clinical parameters will be taken and recorded:

Blood pressure, cardiac frequency and electrocardiogram.

Both an urine and a blood (8 ml) sample will be taken for assays routinely performed in the follow up of diabetes therapy:

Routine blood assay:

- Hemochrome (including White Cells)
- Glucose
- Glycated hemoglobin (HbA1c)
- Creatinine
- Urea
- Glutamic-oxalacetic transaminase (GOT)
- Glutamic-pyruvic transaminase (GPT)
- Alcaline phosphatase
- Triglicerids
- Total cholesterol (t-CH)
- Low-Density Lipoproteins (LDL-CH)
- High-Density Lipoproteins (HDL-CH)
- Apo-protein AI (Apo-AI)
- Apo-protein B (Apo-B)
- Apo-protein C-III (Apo-CIII)
- Eritrosedimentation speed (ES)
- hsCRP
- Endothelin 1 (ET-1)

Urine assay:

- Microalbuminuria
- Macroalbuminuria
- Physical and chemical urine examination

VISIT 8 (week 24, last visit of the treatment phase):
The patient will return the diary form and the empty medication boxes/blister. The investigator will interview the patient and the following clinical parameters will be taken and recorded:

Blood pressure, cardiac frequency and electrocardiogram, weight and waist/hip ratio and andrological examination, the latter one consisting in objective clinical evaluation of both volume and consistency of breast, testes and penis.

Both an urine and a blood (8 ml) sample will be taken for assays routinely performed in the follow up of diabetes therapy:

Routine blood assay:

- Hemochrome (including White Cells)
- Glucose
- Glycated hemoglobin (HbA1c)
- Creatinine
- Urea
- Glutamic-oxalacetic transaminase (GOT)
- Glutamic-pyruvic transaminase (GPT)
- Alcaline phosphatase
- Triglicerids
- Total cholesterol (t-CH)
- Low-Density Lipoproteins (LDL-CH)
- High-Density Lipoproteins (HDL-CH)
- Apo-protein AI (Apo-AI)
- Apo-protein B (Apo-B)
- Apo-protein C-III (Apo-CIII)
- Eritrosedimentation speed (ES)
- hsCRP
- Endothelin 1 (ET-1)

Urine assay:

- Microalbuminuria
- Macroalbuminuria
- Physical and chemical urine examination

Two further blood samples (8 ml in total) will be taken for storage of plasma and serum (–20°C) for the measurement of sexual hormones and of parameters of endothelial function to be performed at the end of the study:

Parameters of endothelial function:

- Interleukin 6 (IL-6)
- Tumor necrosis factor alfa (TNF-alfa)
- Von Willebrand factor (vWF)
- Fibrinogen
- Plasminogen activator 1 (PAI-1)
- Homocysteine
- Adiponectin

Sexual hormones:

- Luteinizing-hormone (LH)
- Follicular-stimulating- hormone (FSH)
- Testosterone
- Estradiol (E2)
- Sex-hormone binding globulin (SHBG)

The following instrumental investigations will be performed:
FMD and PWV.

In addition the patient will undergo an unstructured sexological interview and provided with questionnaires to be filled during the visit: the self-filled questionnaire “International Index of Erectile Function” (Rosen et al. 1997) and the self-filled questionnaire “Beck’s Depression Inventory” (Beck et al. 1961).
After this visit, the patient will enter the follow up phase.

4.8 Follow up phase
This is a medication-free phase in which we should observe a return to baseline levels of the primary end point. However, it is difficult to predict the time necessary for serum endothelin 1 concentrations to return to baseline and this parameter will not be measured until the end of the study. In a previous study Rosano et al. (2005) observed a sustained improvement of FMD and serum endothelin 1 levels which lasted at least two weeks after discontinuation of study medication (tadalafil, 20 mg on alternate days for four weeks). The follow up phase of our study will last at least 24 weeks. Patients will be seen in two visits, i.e. at study week 28 and 48.

VISIT 9 (study week 28, 4 weeks after treatment discontinuation):
The investigator will interview the patient and the following clinical parameters will be taken and recorded:

Blood pressure, cardiac frequency and electrocardiogram.

Both an urine and a blood (8 ml) sample will be taken for assays routinely performed in the follow up of diabetes therapy:

Routine blood assay:

- Hemochrome (including White Cells)

Glucose
Glycated hemoglobin (HbA1c)
Creatinine
Urea
Glutamic-oxalacetic transaminase (GOT)
Glutamic-pyruvic transaminase (GPT)
Alcaline phosphatase
Triglicerids
Total cholesterol (t-CH)
Low-Density Lipoproteins (LDL-CH)
High-Density Lipoproteins (HDL-CH)
Apo-protein AI (Apo-AI)
Apo-protein B (Apo-B)
Apo-protein C-III (Apo-CIII)
Eritrosedimentation speed (ES)
hsCRP
Endothelin 1 (ET-1)

Urine assay:

Microalbuminuria
Macroalbuminuria
Physical and chemical urine examination

Two further blood samples (8 ml in total) will be taken for storage of plasma and serum (–20°C) for the measurement of parameters of endothelial function to be performed at the end of the study:

Parameters of endothelial function:

Interleukin 6 (IL-6)
Tumor necrosis factor alfa (TNF-alfa)
Von Willebrand factor (vWF)
Fibrinogen
Plasminogen activator 1 (PAI-1)
Homocysteine
Adiponectin

FINAL VISIT (Visit 10, study week 48, 24 weeks after treatment discontinuation):

This is the last visit of the study. The investigator will interview the patient and the following clinical parameters will be taken and recorded:

Blood pressure, cardiac frequency and electrocardiogram, weight and waist/hip ratio and andrological examination, the latter one consisting in objective clinical evaluation of both volume and consistency of breast, testes and penis.

Both an urine and a blood (8 ml) sample will be taken for assays routinely performed in the follow up of diabetes therapy:

Routine blood assay:

- Hemochrome (including White Cells)
- Glucose
- Glycated hemoglobin (HbA1c)
- Creatinine
- Urea
- Glutamic-oxalacetic transaminase (GOT)
- Glutamic-pyruvic transaminase (GPT)
- Alcaline phosphatase
- Triglicerids
- Total cholesterol (t-CH)
- Low-Density Lipoproteins (LDL-CH)
- High-Density Lipoproteins (HDL-CH)
- Apo-protein AI (Apo-AI)
- Apo-protein B (Apo-B)
- Apo-protein C-III (Apo-CIII)
- Eritrosedimentation speed (ES)
- hsCRP
- Endothelin 1 (ET-1)

Urine assay:

- Microalbuminuria
- Macroalbuminuria
- Physical and chemical urine examination

Two further blood samples (8 ml in total) will be taken for storage of plasma and serum (–20°C) for the measurement of sexual hormones and of parameters of endothelial function to be performed at the end of the study:

Parameters of endothelial function:

Interleukin 6 (IL-6)
Tumor necrosis factor alfa (TNF-alfa)
Von Willebrand factor (vWF)
Fibrinogen
Plasminogen activator 1 (PAI-1)
Homocysteine
Adiponectin

Sexual hormones:

Luteinizing-hormone (LH)
Follicular-stimulating- hormone (FSH)
Testosterone
Estradiol (E2)
Sex-hormone binding globulin (SHBG)

The following instrumental investigations will be performed:
FMD and PWV.

In addition the patient will undergo an unstructured sexological interview and provided with questionnaires to be filled during the visit: the self-filled questionnaire “International Index of Erectile Function” (Rosen et al. 1997) and the self-filled questionnaire “Beck’s Depression Inventory” (Beck et al. 1961).

4.9 Adverse events

An independent Data Safety and Monitoring Committee (DSMC), comprised of the trial monitor and 2 external experts, will be established. The Committee members will provide expertise in clinical medicine related to diabetes, male endocrinology, ethics, statistics, and safety/pharmacology. The DSMC will operate according to standard rules of good clinical practice, including scientific integrity and confidentiality of the data and reports. The DSMC will meet once for an interim analysis (when 50% of the patients have completed visit 5) and at the end of the study or earlier if needed (for instance in case of serious adverse event or if specifically required by the Principal Investigator (PI) at his discretion) and will:

review results from interim analysis of the accumulating data (if available);
examine efficacy and safety data according to the predefined schedule;

monitor the quality of the trial data;

deliberate and give guidance on any ethical or unexpected safety and/or efficacy issues that may arise during the trial;

make recommendations to the PI concerning continuation or termination of the study or any other modification of the study, based on the observed beneficial or adverse effects of the study medication.

4.10 Criteria for discontinuation

4.10.1 Of individual subjects

Any study patient can terminate his participation at any time, for any reason, and without giving a reason. The PI can discontinue a given participant if, in his opinion, the patient no longer meets the inclusion/exclusion criteria or if the clinical observations during the study suggest that it might be unsafe for that participant to continue. Possible circumstances for study termination for a particular participant may include:

Medical prudence, at the discretion of the Investigator

Poor compliance or cooperation of the patient, as judged by the Investigator

Non-reporting to the centre within ± 5 days of the scheduled appointment for administration of the study compounds

Two consecutive missed visits

Use of medicinal products or drugs not permitted during the study (to be indicated in Investigator's Brochure)

Serious adverse event related to study product

Participant’s withdrawal due to adverse event

Occurrence of a medical condition requiring exclusion from the study (e.g. cardiovascular disease necessitating NO donors therapy)

Pathologically changed laboratory values

Suspected drug interaction

Other non-study related reasons: e.g. moving out of the study area

Revocation of consent to participate in the study

If the study is discontinued prematurely for any participant because of a serious adverse event, the information listed below should be documented, with copies of all associated evaluations and laboratory studies:

Date and, if applicable, time of last administration of investigational products

Date of Investigator's last contact with the subject, if applicable

Every effort will be made to follow all study participants until the end of the study. Reasons for discontinuation of the Follow up Phase include:

Loss to follow-up

Subject's expressed desire to discontinue participation

4.10.2 Of the study

The following are proposed criteria for study discontinuation:

- unacceptable rates of side effects
- any other reason that indicates that continuation might compromise the health or well being of participating patients

4.11 Laboratory and other investigations

After an overnight fast the first urines will be collected; furthermore an i.v. cannula will be inserted into an antecubital vein in order to collect a blood sample for serum and plasma assays. Blood samples for immediate analysis will be forwarded to the laboratory while the blood samples for analysis at the end of the study will be centrifuged and the serum/plasma stored at – 20° C until assayed. The total amount of blood taken from each subject will be a maximum of 150-200 ml over the entire study period.

The **blood (serum/plasma) and urinary parameters** routinely assessed in the follow up of diabetes on antidiabetic treatments (hemochrome (including White Cells), glucose, glycated hemoglobin (HbA1c), creatinine, urea, GOT, GPT, alcaline phosphatase, triglicerids, total cholesterol (t-CH), LDL-CH, HDL-CH, Apo-AI, Apo-B, Apo-CIII, eritrosedimentation speed and hsCRP, micro- and macroalbuminuria, physical and chemical urine examination) will be measured on real time by the central laboratory according to their standard procedures.

The parameters to be measured at the end of the study include: ET-1, IL-6, TNF-alfa, vWF, fibrinogen, PAI-1, Homocysteine, Adiponectin, ,LH, FSH, testosterone, estradiol, SHBG. These parameters will be assayed by using commercially available kits at the Research Laboratory of the Department.

Questionnaires

The subjects will be asked to fill the two following questionnaires at baseline (visit 1), after 8 weeks (visit 5), 24 weeks (visit 8) and 48 weeks (study end, visit 10):

International Index of Erectile Function (Rosen et al. 1997), a self-filled 15 questions questionnaire consistent in 5 domains: orgasm (questions 9, 10; score range 0 – 10), sexual desire (questions 11, 12; score range 2 – 10), intercourse satisfaction (questions 6 - 8; score range 0 – 15), general

wellbeing (questions 13, 14; score range 2 – 10), erectile function (questions 1 - 5, 15; score range 1 – 30).

For all the 5 domains a high score indicates normality of the investigated parameters. Only the erectile function domain has a detailed score interpretation: a score less than or equal to 10 indicates severe ED;11 to 16, moderate ED; 17 to 25, light ED; more than or equal to 26, absent ED.

Beck's Depression Inventory (Beck et al. 1961), a self-filled 21 questions questionnaire (score range 0 – 63); a score less than or equal to 9 indicates no depression; 10 to 19, mild to moderate depression; more than or equal to 20, clinically significant depression.

Instrumental investigations

The following investigations will be performed at week 1 (visit 2), week 24 (visit 8) and at the end of the study, week 48 (visit 10), in order to evaluate the presence and the severity of macrovascular and coronary artery disease:

Brachial artery flow-mediated dilation (FMD), which is an endothelial dependent dilation; this parameter is assested by measuring the change in brachial artery diameter after 60 sec. of reactive hyperemia compared with baseline measurements after deflation of a cuff placed around the forearm that had been inflated to 50 mm Hg above systolic blood pressure for 5 minutes (Corretti et al. 2002).

Pulse wave velocity (PWV), which is the velocity of propagation of the arterial blood pressure wave along the vascular wall. PWV serves as an index to evaluate the stiffness of the vessels and the after-load of the heart, and as a marker related to motional capacity. The pulse wave is more rapidly transmitted when the inner diameter of the artery is thinner; when its wall is thicker, its extendibility is poorer. The higher the blood pressure, the higher the tension of the vascular wall, and the less the extendibility, resulting in a higher PWV. It has been reported that PWV is, at least in part, a marker of the severity of arteriosclerosis-related vascular damage. It has been shown in many studies that the values of PWV in subjects with hypertension and diabetes are higher than those in normal subjects (Laurent et al. 2001).

4.12 Data management and analysis

The study will be conducted and monitored according to Good Clinical Practice requirements. Appropriate data forms (Case Report Forms: CRF) will be developed and kept updated with all clinical data by the study personnel. A trial auditor will be designed at the beginning of the study to

review the CRFs and the clinical data at regular intervals. The personnel involved will allow the study auditor, to monitor the course of the clinical trial. On these occasions, the auditor will check the CRFs for completeness and correctness by comparing them with the source data of the subjects (Source Data Verification). The practice/clinic and laboratory facilities may likewise be inspected.

A trial manager will be designed at the beginning of the study and will be responsible for reviewing and checking monitored data and for raising queries on inconsistencies or unexpected values that have not been addressed previously. The study manager will also be responsible for analyzing data, generating tabulated data and for preparation of the final study report.

Data collection is the responsibility of the clinical trial staff under the supervision of the Principal Investigator.

Data will be analysed by descriptive statistics, ANCOVA and multiple regression analysis or non-parametric tests as appropriate by STATA. Data stratification by selected background characteristics might be performed if appropriate. Appropriate transformation of non-normal or skewed measurement will be performed on continuous measurement and mean and standard deviations will be reported overall and by background characteristics. Number and percentages will be used to summarize categorical measurements.

An interim analysis will be conducted if requested by the Data Safety and Monitoring Committee.

The Principal Investigator is responsible for maintaining complete and accurate study documentation for the study.

4.13 Trial registration

The trial will be registered at http://oss-sper-clin.agenziafarmaco.it/ and an European EUDRACT registration number will be obtained.

4.14 Insurance and ethics committee

Ethics committee approval and patient informed consent will be obtained and patients will be appropriately insured.

5. Expected outcome

According to the working hypothesis, we expect an increased protective effect of NO on the endothelium as a result of the increase of the cellular levels of cGMP due to PDE5 inhibition only in

the verum group. This will be reflected by reduced levels of vascular inflammation markers, (endothelin 1 and possibly other markers of inflammation), improvement (or no worsening) of PWV and FMD. In addition, according to the experimental data of the literature, vardenafil treatment might improve (or reduce worsening) microalbuminuria (i.e. microangiopathy) and possibly even glycemic control, as documented by HbA1c levels.

An easily foreseeable outcome should be a significant improvement, in the verum group, of sexual activity in terms of penile erection and possibly of both sexual interest and sexual activity frequency, even if these last two effects should be regarded as an indirect effect due to better erections.

No major adverse events (see 6) are anticipated.

6. Main problems anticipated

No specific technical problems are anticipated. Common side effects of vardenafil (headache, flushing, rhinitis, dyspepsia, sinusitis and flu-syndrome, dizziness and nausea) are not expected to occur more frequently in this category of patients.

Poor compliance might be a problem in patients with type 2 diabetes mellitus. This justifies the high frequency of visits throughout the study.

There may be a higher-than-anticipated rate of discontinuation from the trial, limiting the statistical power in the final analysis.

7. Adverse events

An adverse event or adverse experience (AE) is any untoward medical occurrence in a clinical investigation subject who has been administered a pharmaceutical product and which does not necessarily have a causal relationship with this treatment. An AE can therefore be any unfavorable and unintended sign (including an abnormal laboratory finding), symptom, or disease temporally associated with the use of an investigational product, whether or not considered related to the investigational product. Pre-existing events that increase in frequency or severity in nature during or as a consequence of use of a drug in human clinical trials will also be considered as adverse events. Any medical condition or clinically significant laboratory abnormality with an onset date before the first date of study drug administration is considered to be pre-existing, and should be documented in the source document for the participant.

The following definitions will be used in this study:

"*Associated with the use of the drug*" means: "there is a reasonable possibility that the experience may have been caused by the drug."

"*Unexpected adverse drug experience*" means: "Any adverse drug experience, the specificity or severity of which is not consistent with the risk information described in the general investigational plan or elsewhere in the current application."

"*Serious adverse drug experience*" (SAE) means: "Any adverse drug experience occurring at any dose that results in any of the following outcomes: Death, a life-threatening adverse drug experience, inpatient hospitalization or prolongation of existing hospitalization, a persistent or significant disability/incapacity, or a congenital anomaly/birth defect. Important medical events that may not result in death, be life-threatening, or require hospitalization may be considered a serious adverse drug experience when, based upon appropriate medical judgment, they may jeopardize the patient or subject and may require medical or surgical intervention to prevent one of the outcomes listed in this definition."

Any adverse events that are serious (whether expected or unexpected) and possibly associated with use of the drug will be immediately reported to the Data Safety and Monitoring Committee and to the Medical Officer of Bayer-Schering by telephone and e-mail.

Adverse events that do not fall into the categories of associated with drug, serious, and unexpected may include illnesses, discomfort, pain, or any medical problem such as cold symptoms or other complaints. All adverse events, whether serious or non-serious and whether or not related to the study drug, should be recorded on the Adverse Event Form. All reports of adverse events will be reviewed and signed by qualified medical personnel.

For each adverse event, an assessment of the relatedness to the test agent should be made using the following scale:

Unrelated: Onset of the AE had no reasonable temporal relationship to administration of the study product or a causal relationship to administration of the study product is biologically implausible or the event is attributed to an alternative etiology.

Possibly Related: Onset of the AE has a reasonable temporal relationship to study product administration and a causal relationship is not biologically implausible.

Probably Related: Onset of the AE has a strong temporal relationship to administration of the study product that cannot be explained by the subject’s clinical state or other factors and a causal relationship is not biologically implausible.

Definitely Related: Onset of the AE shows a distinct temporal relationship to administration of the study product that cannot be explained by the subject’s clinical state or other factors or the AE occurs on rechallenge or the AE is a know reaction to the product or chemical group or can be predicted by the product’s pharmacology.

Each adverse event should be graded for severity using the following scale:

Mild: the participant was aware of the adverse event, but was still able to do all activities; no or minimal medical intervention/therapy required.

Moderate: the participant had to discontinue some activities due to the adverse event; no or minimal medical intervention/therapy required.

Severe: the participant was incapacitated by the adverse event and unable to perform most normal activities; significant medical intervention/therapy required, hospitalization possible.

8. References

Ahn GJ, Yu JY, Choi SM, Kang KK, Ahn BO, Kwon JW, Kang SK, Lee BC, Hwang WS. Chronic administration of phosphodiesterase 5 inhibitor improves erectile and endothelial function in a rat model of diabetes. Int J Androl. 2005;28:260-266.

Aversa A, Greco E, Bruzziches R, Pili M, Rosano G, Spera G. Relationship between chronic tadalafil administration and improvement of endothelial function in men with erectile dysfunction: a pilot study. Int J Impot Res. 2007;19:200-207.

Aversa A, Vitale C, Volterrani M, Fabbri A, Spera G, Fini M, Rosano GM. Chronic administration of Sildenafil improves markers of endothelial function in men with Type 2 diabetes. Diabet Med. 2008;25:37-44.

Basu A, Ryder RE. New treatment options for erectile dysfunction in patients with diabetes mellitus. Drugs. 2004;64:2667-2688.

Beck AT, Ward CH, Mendelson M, Mock J, Erbaugh J. An inventory for measuring depression. Arch Gen Psychiatry. 1961;4:53-63.

Beller EM, Gebski V, Keech AC. Randomisation in clinical trials. Med J Aust. 2002;177:565-567

Bender AT, Beavo JA. Cyclic nucleotide phosphodiesterases: molecular regulation to clinical use. Pharmacol Rev. 2006;58:488-520.

Bugiardini R, Manfrini O, Pizzi C, Fontana F, Morgagni G. Endothelial function predicts future development of coronary artery disease: a study of women with chest pain and normal coronary angiograms. Circulation. 2004;109:2518-2523.

Chiurlia E, D'Amico R, Ratti C, Granata AR, Romagnoli R, Modena MG. Subclinical coronary artery atherosclerosis in patients with erectile dysfunction. J Am Coll Cardiol. 2005;46:1503-1506.

Clapp BR, Hingorani AD, Kharbanda RK, Mohamed-Ali V, Stephens JW, Vallance P, MacAllister RJ. Inflammation-induced endothelial dysfunction involves reduced nitric oxide bioavailability and increased oxidant stress. Cardiovasc Res. 2004;64:172-178.

Corbin JD. Mechanisms of action of PDE5 inhibition in erectile dysfunction. Int J Impot Res. 2004;16(1):4-7.

Corretti MC, Anderson TJ, Benjamin EJ, Celermajer D, Charbonneau F, Creager MA, Deanfield J, Drexler H, Gerhard-Herman M, Herrington D, Vallance P, Vita J, Vogel R. Guidelines for the ultrasound assessment of endothelial-dependent flow-mediated vasodilation of the brachial artery: a report of the International Brachial Artery Reactivity Task Force. J Am Coll Cardiol. 2002;39:257-265.

Cosentino F, Lüscher TF. Endothelial dysfunction in diabetes mellitus. J Cardiovasc Pharmacol. 1998;32:54-61.

Creager MA, Cooke JP, Mendelsohn ME, Gallagher SJ, Coleman SM, Loscalzo J, Dzau VJ. Impaired vasodilation of forearm resistance vessels in hypercholesterolaemic humans. J Clin Invest. 1990;86:228-234.

Creager MA, Lüscher TF, Cosentino F, Beckman JA. Diabetes and vascular disease: pathophysiology, clinical consequences, and medical therapy: Part I. Circulation. 2003;108:1527-1532.

De Young LX, Domes T, Lim K, Carson J, Brock GB. Endothelial rehabilitation: the impact of chronic PDE5 inhibitors on erectile function and protein alterations in cavernous tissue of diabetic rats. Eur Urol. 2008;54:213-220.

Desouza C, Parulkar A, Lumpkin D, Akers D, Fonseca VA. Acute and prolonged effects of sildenafil on brachial artery flow-mediated dilation in type 2 diabetes. Diabetes Care. 2002;25:1336-1339.

Duckworth W, Abraira C, Moritz T, Reda D, Emanuele N, Reaven PD, Zieve FJ, Marks J, Davis SN, Hayward R, Warren SR, Goldman S, McCarren M, Vitek ME, Henderson WG, Huang GD; VADT Investigators. Glucose control and vascular complications in veterans with type 2 diabetes. N Engl J Med. 2009;360:129-139.

Dunkern TR, Hatzelmann A. The effect of sildenafil on human platelet secretory function is controlled by a complex interplay between phosphodiesterases 2, 3 and 5. Cell Signal. 2005;17:331-339.

European Association for the Study of Diabetes Type 2 diabetes mellitus guidelines. European Heart Journal. 2007;28:88-136.

Ferri C, Pittoni V, Piccoli A, Laurenti O, Cassone MR, Bellini C, Properzi G, Valesini G, De Mattia G, Santucci A. Insulin stimulates endothelin 1 secretion from human endothelial cells and modulates its circulating levels in vivo. J Clin Endocrinol Metab. 1995;80:829-835.

Gazzaruso C, Giordanetti S, De Amici E, Bertone G, Falcone C, Geroldi D, Fratino P, Solerte SB, Garzaniti A. Relationship between erectile dysfunction and silent myocardial ischemia in apparently uncomplicated type 2 diabetic patients. Circulation. 2004;110:22-26.

Grover-Páez F, Villegas Rivera G, Guillén Ortíz R. Sildenafil citrate diminishes microalbuminuria and the percentage of A1c in male patients with type 2 diabetes. Diabetes Res Clin Pract. 2007;78:136-140.

Hatzichristou D, Gambla M, Rubio-Aurioles E, Buvat J, Brock GB, Spera G, Rose L, Lording D, Liang S. Efficacy of tadalafil once daily in men with diabetes mellitus and erectile dysfunction. Diabet Med. 2008;25:138-146.

Kapur V, Chien CV, Fuess JE, Schwarz ER. The relationship between erectile dysfunction and cardiovascular disease. Part II: The role of PDE-5 inhibition in sexual dysfunction and cardiovascular disease. Rev Cardiovasc Med. 2008;9:187-195.

Kataoka C, Egashira K, Inoue S, Takemoto M, Ni W, Koyanagi M, Kitamoto S, Usui M, Kaibuchi K, Shimokawa H, Takeshita A. Important role of Rho-kinase in the pathogenesis of cardiovascular inflammation and remodelling induced by long-term blockade of nitric oxide synthesis in rats. Hypertension. 2002;39:245-250.

Kearney MT, Duncan ER, Kahn M, Wheatcroft SB. Insulin resistance and endothelial cell dysfunction: studies in mammalian models. Exp Physiol. 2008;93:158-163.

Ito M, Nishikawa M, Fujioka M, Miyahara M, Isaka N, Shiku H, Nakano T. Characterization of the isoenzymes of cyclic nucleotide phosphodiesterase in human platelets and the effects of E4021. Cell Signal. 1996;8:575-581.

Lau DH, Mikhailidis DP, Thompson CS. The effect of vardenafil (a PDE type 5 inhibitor) on renal function in the diabetic rabbit: a pilot study. In Vivo. 2007;21:851-854.

Laurent S, Boutouyrie P, Asmar R, Gautier I, Laloux B, Guize L, Ducimetiere P, Benetos A. Aortic stiffness is an independent predictor of all-cause and cardiovascular mortality in hypertensive patients. Hypertension. 2001;37:1236-1241.

Levien TL. Phosphodiesterase inhibitors in Raynaud's phenomenon. Ann Pharmacother. 2006;40:1388-1393.

Lin CS, Chow S, Lau A, Tu R, Lue TF. Human PDE5A gene encodes three PDE5 isoforms from two alternate promoters. Int J Impot Res. 2002;14:15-24.

Lubos E, Handy DE, Loscalzo J. Role of oxidative stress and nitric oxide in atherothrombosis. Front Biosci. 2008;13:5323-5344.

Lue T, Goldstein I, Traish A. Comparison of oral and intracavernosal vasoactive agents in penile erection. Int J Impot Res. 2000;12:81-88.

Mather KJ, Verma S, Anderson TJ. Improved endothelial function with metformin in type 2 diabetes mellitus. J Am Coll Cardiol. 2001;37:1344-1350.

Ministero della Salute, Relazione sullo stato sanitario del paese, 2000. http://www.ministerosalute.it/dettaglio/pdPrimoPiano.jsp?sub=1&id=107&area=ministero%09%09&colore=2&lang=it.

Moncada S, Higgs A. The L-arginine-nitric oxide pathway. N Engl J Med. 1993;329:2002-2012.

NIH Consensus Conference. Impotence. JAMA. 1993;270:83-90.

Piatti PM, Monti LD, Conti M, Baruffaldi L, Galli L, Phan CV, Guazzini B, Pontiroli AE, Pozza G. Hypertriglyceridemia and hyperinsulinemia are potent inducers of endothelin 1 release in humans. Diabetes. 1996; 45:316-321.

Prickaerts J, Sik A, van Staveren WC, Koopmans G, Steinbusch HW, van der Staay FJ, de Vente J, Blokland A. Phosphodiesterase type 5 inhibition improves early memory consolidation of object information. Neurochem Int. 2004;45:915-928.

Richardson D, Vinik A. Etiology and treatment of erectile failure in diabetes mellitus. Curr Diab Rep. 2002;2:501-509.

Rosano GM, Aversa A, Vitale C, Fabbri A, Fini M, Spera G. Chronic treatment with tadalafil improves endothelial function in men with increased cardiovascular risk. Eur Urol. 2005;47:214-220.

Rosen RC, Riley A, Wagner G, Osterloh IH, Kirkpatrick J, Mishra A. The International Index of Erectile Function (IIEF): a multidimensional scale for assessment of erectile dysfunction. Urology. 1997;49:822-830.

Rosen RC, Kostis JB. Overview of phosphodiesterase 5 inhibition in erectile dysfunction. Am J Cardiol. 2003;92:9-18.

Schachinger V, Britten MB, Zeiher AM. Prognostic impact of coronary vasodilator dysfunction on adverse long-term outcome of coronary heart disease. Circulation 2000;101:1899-1906.

Schäfer A, Fraccarollo D, Pförtsch S, Flierl U, Vogt C, Pfrang J, Kobsar A, Renné T, Eigenthaler M, Ertl G, Bauersachs J. Improvement of vascular function by acute and chronic treatment with the PDE-5 inhibitor sildenafil in experimental diabetes mellitus. Br J Pharmacol. 2008;153:886-893.

Schäfer A, Wiesmann F, Neubauer S, Eigenthaler M, Bauersachs J, Channon KM. Rapid regulation of platelet activation in vivo by nitric oxide. Circulation. 2004;109:1819-1822.

Setter SM, Iltz JL, Fincham JE, Campbell RK, Baker DE. Phosphodiesterase 5 inhibitors for erectile dysfunction. Ann Pharmacother. 2005;39:1286-1295.

Sommer F, Klotz T, Engelmann U. Improved spontaneous erectile function in men with mild-to-moderate arteriogenic erectile dysfunction treated with a nightly dose of sildenafil for one year: a randomized trial. Asian J Androl. 2007;9:134-141.

Sommer F, Schulze W. Treating erectile dysfunction by endothelial rehabilitation with phosphodiesterase 5 inhibitors. World J Urol. 2005;23:385-392.

Takimoto E, Champion HC, Li M, Belardi D, Ren S, Rodriguez ER, Bedja D, Gabrielson KL, Wang Y, Kass DA. Chronic inhibition of cyclic GMP phosphodiesterase 5A prevents and reverses cardiac hypertrophy. Nat Med. 2005;11:214-222.

Tanner FC, Meier P, Greutert H, Champion C, Nabel EG, Lüscher TF. Nitric oxide modulates expression of cell cycle regulatory proteins: a cytostatic strategy for inhibition of human vascular smooth muscle cell proliferation. Circulation. 2000;101:1982-1989.

The Action to Control Cardiovascular Risk in Diabetes Study Group. Effects of intensive glucose lowering in type 2 diabetes. N Engl J Med 2008;358:2545-2559.

The ADVANCE Collaborative Group. Intensive blood glucose control and vascular outcomes in patients with type 2 diabetes. N Engl J Med 2008;358:2560-2572.

Timimi FK, Ting HH, Haley EA, Roddy MA, Ganz P, Creager MA. Vitamin C improves endothelium-dependent vasodilation in patients with insulin-dependent diabetes mellitus. J Am Coll Cardiol. 1998;31:552-557.

Ting HH, Timimi FK, Boles KS, Creager SJ, Ganz P, Creager MA. Vitamin C improves endothelium-dependent vasodilation in patients with non–insulin-dependent diabetes mellitus. J Clin Invest. 1996;97:22-28.

Vardi M, Nini A. Phosphodiesterase inhibitors for erectile dysfunction in patients with diabetes mellitus. Cochrane Database Syst Rev. 2007;24:CD002187.

Wild S, Roglic G, Green A, Sicree R, King H. Global prevalence of diabetes: estimates for the year 2000 and projections for 2030. Diabetes Care. 2004;27:1047-1053.

Zhang C. The role of inflammatory cytokines in endothelial dysfunction. Basic Res Cardiol. 2008;103:398-406.

9. The study at a glance (synoptic table)

		Treatment phase								
	Enrolment phase								Follow-up phase	
	Visit 1	Visit 2 week 1	Visit 3 week 2	Visit 4 week 4	Visit 5 week 8	Visit 6 week 12	Visit7 week 18	Visit 8 week 24	Visit 9 week 28	Visit 10 week 48
Screening	X	X								
Consent		X								
Starting protocol		X								
Anamnesis	X									
Physical exam.[1]	X			X		X		X		X
Andrological exam.[2]	X							X		X
Cardiovascular exam.[3]	X		X	X	X	X	X	X	X	X
Questionnaires[4]	X				X			X		X
Routine collection[5]	X		X	X	X	X	X	X	X	X
Instrumental exam.[6]		X						X		X
Urine collection[7]	X		X	X	X	X	X	X	X	X
Endothelial coll.[8]		X	X	X		X		X	X	X
Endocrine collection[9]		X						X		X

[1]Physical examination: weight, waist/hip ratio, height (height only at visit 1)
[2]Andrological examination: visual/tactile exam of testes and penis
[3]Cardiovascular examination: blood pressure, cardiac frequency, ECG
[4]Questionnaires: International Index of Erectile Function and Beck's Depression Inventory
[5]Blood collection for routine assays: hemochrome, glucose, HbA1c, creatinine, urea, GOT, GPT, alkaline phosphatise, triglycerids, total cholesterol, LDL, HDL, Apo-AI, Apo-B, Apo-CIII, eritrosedimentation speed, CRP, endothelin 1
[6]Instrumental examinations: Flow Mediated Dilation and Pulse Wave Velocity
[7]Urine collection: microalbuminuria, macroalbuminuria, physical and chemical urine examination
[8]Blood collection for endothelial function parameters: IL-6, TNF-α, von-Willebrand factor, fibrinogen, PAI-1, homocysteine, adiponectin
[9]Blood collection for sexual hormones: LH, FSH, testosterone, estradiol, SHBG
