# Supplementary material for: Could chronic Vardenafil administration influence the cardiovascular risk in men with type 2 diabetes mellitus?
Source: PLoS One. 2018 Jun 28;13(6):e0199299. doi: 10.1371/journal.pone.0199299 (PMC6023114; doi:10.1371/journal.pone.0199299)
Supplement: S3 File — (PDF) [file pone.0199299.s003.pdf]

Pz nr.	Visita	Tp pressione sì(1) no(0)	Vardenafil	Hypogonadism
1	2	0	**1**	0,000
2	2	0	**0**	0,000
3	2	0	**0**	0,000
4	2	1	**1**	0,000
5	2	1	**1**	1,000
6	2	1	**0**	0,000
7	2	1	**1**	1,000
8	2	1	**0**	0,000
9	2	0	**1**	0,000
10	2	0	**0**	0,000
11	2	1	**1**	0,000
12	2	0	**0**	1,000
13	2	0	**0**	0,000
14	2	1	**1**	0,000
15	2	0	**1**	0,000
16	2	1	**0**	1,000
17	2	0	**1**	0,000
18	2	1	**0**	0,000
19	2	1	**1**	1,000
20	2	1	**0**	1,000
21	2	0	**0**	0,000
22	2	0	**1**	1,000
23	2	0	**0**	0,000
24	2	1	**1**	1,000
25	2	1	**1**	0,000
26	2	1	**0**	0,000
27	2	1	**0**	1,000
28	2	1	**1**	0,000
29	2	1	**0**	0,000
30	2	1	**1**	0,000
31	2	0	**0**	0,000
32	2	0	**0**	0,000
33	2	0	**1**	0,000
34	2	0	**1**	0,000
35	2	1	**0**	0,000
36	2	1	**0**	0,000
37	2	1	**1**	0,000
38	2	1	**1**	1,000
39	2	1	**0**	0,000
40	2	0	**0**	0,000
41	2	0	**1**	0,000
42	2	0	**0**	0,000
43	2	0	**1**	0,000
44	2	0	**1**	0,000
45	2	0	**1**	0,000

46	2	0	**0**	0,000
47	2	0	**0**	0,000
48	2	1	**0**	0,000
49	2	0	**1**	1,000
50	2	1	**1**	0,000
51	2	0	**1**	0,000
52	2	1	**0**	1,000
53	2	0	**0**	1,000
54	2	0	**0**	0,000
2	8	0	**0**	0,000
3	8	1	**0**	0,000
4	8	1	**1**	0,000
5	8	1	**1**	1,000
6	8	1	**0**	0,000
8	8	0	**0**	0,000
9	8	0	**1**	0,000
10	8	0	**0**	0,000
11	8	1	**1**	0,000
13	8	1	**0**	0,000
14	8	1	**1**	0,000
15	8	0	**1**	0,000
16	8	1	**0**	1,000
17	8	0	**1**	0,000
18	8	1	**0**	0,000
19	8	1	**1**	1,000
21	8	0	**0**	0,000
22	8	0	**1**	1,000
23	8	0	**0**	0,000
24	8	1	**1**	1,000
25	8	1	**1**	0,000
26	8	1	**0**	0,000
27	8	1	**0**	1,000
28	8	1	**1**	0,000
29	8	1	**0**	0,000
31	8	0	**0**	0,000
32	8	0	**0**	0,000
35	8	1	**0**	0,000
36	8	1	**0**	0,000
37	8	1	**1**	0,000
38	8	1	**1**	1,000
39	8	1	**0**	0,000
40	8	0	**0**	0,000
42	8	0	**0**	0,000
43	8	0	**1**	0,000
44	8	0	**1**	0,000
45	8	0	**1**	0,000
46	8	0	**0**	0,000
47	8	0	**0**	0,000
48	8	1	**0**	0,000

49	8	0	**1**	1,000
50	8	1	**1**	0,000
51	8	0	**1**	0,000
52	8	1	**0**	1,000
53	8	0	**0**	1,000
2	10	0	**0**	0,000
3	10	1	**0**	0,000
4	10	1	**1**	0,000
5	10	1	**1**	1,000
6	10	1	**0**	0,000
8	10	0	**0**	0,000
9	10	0	**1**	0,000
10	10	0	**0**	0,000
11	10	1	**1**	0,000
13	10	1	**0**	0,000
14	10	1	**1**	0,000
15	10	0	**1**	0,000
16	10	1	**0**	1,000
17	10	0	**1**	0,000
18	10	1	**0**	0,000
19	10	1	**1**	1,000
21	10	0	**0**	0,000
22	10	0	**1**	1,000
23	10	0	**0**	0,000
24	10	1	**1**	1,000
25	10	1	**1**	0,000
26	10	1	**0**	0,000
27	10	1	**0**	1,000
28	10	1	**1**	0,000
29	10	1	**0**	0,000
31	10	0	**0**	0,000
32	10	0	**0**	0,000
35	10	1	**0**	0,000
36	10	1	**0**	0,000
37	10	1	**1**	0,000
38	10	1	**1**	1,000
39	10	1	**0**	0,000
40	10	0	**0**	0,000
42	10	0	**0**	0,000
43	10	0	**1**	0,000
44	10	0	**1**	0,000
45	10	0	**1**	0,000
46	10	0	**0**	0,000
47	10	0	**0**	0,000
48	10	1	**0**	0,000
49	10	0	**1**	1,000
50	10	1	**1**	0,000
51	10	0	**1**	0,000
52	10	1	**0**	1,000
53	10	0	**0**	1,000

Mesi DM	Testosterone	IL-6 pg/ml	hCRP mg/dl	ET1
12	13,960	2,900	0,16	
13	12,180	1,900	0,42	
18	19,200	3,080	0,16	9,23
3	18,160	8,600	1,68	4,35
12	8,580	1,900	0,27	
58	15,390	3,890	0,13	
24	8,990	5,770	0,17	
5	16,320	1,900	0,12	
2	20,380	8,800	0,12	
24	19,990	2,950	0,08	
18	15,800	3,610	0,07	
4	9,650	4,770	0,16	
12	18,100	3,680	0,34	
24	22,650	7,690	0,08	4,35
8	12,240	4,980	0,06	
23	7,130	4,030	0,19	
18	19,150	4,490	0,09	
1	13,560	3,530	0,49	
26	5,640	4,280	0,15	1,75
24	8,810	4,530	0,09	
36	12,580	5,840	0,12	3,71
20	7,260	4,410	0,01	6,41
60	22,210	4,010	0,4	
48	8,290	3,560	0,08	
10	10,940	4,410	0,09	3,71
27	14,130	5,310	0,12	
36	5,710	5,000	0,31	
20	23,910	3,450	0,25	
24	20,020	4,640	0,05	
58	15,170	5,260	0,23	
24	20,590	1,900	0,06	
36	19,760	3,780	0,08	
12	16,830	3,450	0,01	
12	17,510	3,920	0,06	
14	17,290	6,780	0,45	
62	12,700	3,350	0,01	
62	13,450	3,350	0,08	
5	6,530	2,440	0,05	
15	12,420	1,900	0,11	
6	13,160	3,780	0,27	80,23
52	13,410	2,430	0,32	
62	14,290	2,380	0,14	
62	16,180	3,180	0,16	
62	12,240	2,610	0,1	
10	15,870	4,200	0,3	

12		15,480	3,880	0,19		
29		12,600	3,590	0,34		
18		10,950	4,270	0,24		
5		10,330	4,240	0,03		
8		14,340	4,500	0,96	9,92	
33		12,620	1,900	0,04	1,88	
57		9,240	2,000	0,03		
26		8,920	1,900	0,4		
49		13,790	2,590	2,76		
	13	21,120	2,080	0,04		
	18	12,840	3,950	0,08		9,23
	3	12,550	4,810	0,62		
	12	10,040	1,900	0,16		
	58	11,160	2,900	0,22		
	5	16,640	2,080	0,14		
	2	1,780	3,080	0,1		
	24	21,220	3,500	0,86		
	18	10,860	1,900	0,06		
	12	13,800	4,300	0,32		
	24	19,080	3,090	0,15		
	8	14,670	2,510	0,05		6,41
	23	9,250	4,310	0,05		
	18	17,050	3,090	0,12		
	1	17,870	2,420	0,19		
	26	15,540	2,550	0,11		
	36	7,140	8,670	0,08		2,83
	20	15,210	3,730	0,01		5,16
	60	31,670	3,900	0,08		3,71
	48	12,930	2,150	0,28		
	10	14,320	3,460	0,06		3,71
	27	13,580	3,060	0,03		
	36	7,310	4,840	0,16		
	20	22,560	1,900	0,28		
	24	14,330	8,320	0,44		
	24	19,490	1,900	0,03		
	36	19,190	1,900	0,01		
	14	13,160	2,540	0,18		
	62	14,260	3,900	0,04		
	62	11,700	1,900	0,05		
	5	17,800	1,900	0,01		
	15	14,410	3,900	0,09		
	6	10,940	6,770	0,27		52,15
	62	15,280	3,900	0,07		
	62	18,790	1,900	0,08		
	62	10,640	2,470	0,06		
	10	17,410	2,090	0,18		
	12	13,960	3,900	0,21		
	29	12,440	3,900	0,28		1,88
	18	8,940	3,780	0,33		

5	10,630	1,900	0,03	
8	9,570	5,220	2,1	9,92
33	3,390	1,900	0,05	
57	9,080	1,800	0,08	
26	2,100	2,300	0,39	1,88
13	15,570	2,180	0,04	
18	11,560	4,350	0,08	
3	11,980	4,100	0,56	
12	6,350	3,600	0,28	
58	13,010	2,330	0,41	
5	14,240	3,620	0,26	
2	11,130	3,560	0,07	
24	17,390	3,250	0,11	
18	13,580	2,690	0,07	
12	18,970	3,190	0,61	
24	18,030	4,690	0,14	
8	10,730	2,590	0,05	
23	10,880	3,510	0,05	
18	13,390	1,900	0,12	
1	12,320	2,420	0,29	
26	10,490	3,280	0,12	4,35
36	8,580	4,170	0,07	
20	10,450	3,710	0,01	
60	22,280	3,370	0,13	4,35
48	8,800	3,480	0,05	9,03
10	14,560	2,490	0,08	
27	12,790	1,900	0,11	
36	5,980	2,540	0,3	
20	22,800	2,760	0,17	5,81
24	14,500	6,270	0,67	
24	17,930	1,900	0,08	1,75
36	20,730	1,900	0,01	3,71
14				
62	14,660	1,900	0,04	
62	19,830	1,900	0,01	
5	7,990	1,900	0,3	
15	14,300	1,900	0,11	
6	13,060	1,900	0,11	
62	16,320	1,900	0,4	
62	15,720	1,900	0,05	
62	12,210	1,900	0,14	
10	16,660	1,900	0,15	
12	11,690	1,900	0,11	
29	13,000	1,900	0,27	
18	10,020	3,130	0,29	
5	9,960	1,900	0,03	44,66
8	9,930	1,900	0,62	
33	4,620	1,900	0,01	
57				
26	2,240	1,900	0,22	

ICAM-1	VCAM-1	Glu 1	HbA1C 1	IIEF 1	IMTDX (cm)
2311800,00	486310,95	132,00	6,90	14,00	0,07
1762000,00	1480600,00	117,00	6,60	21,00	0,08
3044300,00	749803,08	118,00	5,50	1,00	0,01
51921000000,00	1150700,00	117,00	6,20	6,00	0,09
2297700,00	716572,09	104,00	9,30	19,00	0,12
1533700,00	609544,71	145,00	7,70	19,00	0,11
51521000000,00	615556,57	131,00	7,30	14	0,08
53777000000,00	483542,99	133,00	8,00	24	0,08
1862200,00	797918,20	81,00	6,50	24	0,1
1836600,00	777357,00	185,00	8,90	16	0,11
1839800,00	812081,17	99,00	5,30	3	0,9
1082000,00	1119100,00	178,00	7,50	24	0,1
2549500,00	918756,83	148,00	6,60	22	0,08
2558400,00	1980400,00	156,00	6,50	4	0,07
1545800,00	941284,40	128,00	6,30	17	0,09
44222000000,00	595470,32	151,00	7,40	2	0,1
1075700,00	529669,46	231,00	7,60	12	0,1
1354900,00	662885,83	132,00	6,50	26	0,07
52217000000,00	446486,45	167,00	9,40	20,00	0,06
1670400,00	899414,47	157,00	8,10	14,00	0,09
38268000000,00	653649,63	77,00	5,20	9,00	0,1
53982000000,00	1056100,00	117,00	5,30	22,00	0,06
1009700,00	655104,57	109,00	6,00	12,00	0,08
1780800,00	630722,49	113,00	6,40	24,00	0,08
2669600,00	691400,87	159,00	6,90	7	0,09
1832300,00	802392,19	119,00	7,10	11	0,12
1458800,00	910957,24	145,00	6,20	11	0,11
990281,89	862752,20	97,00	5,80	24	0,12
1089400,00	1622100,00	111,00	6,20	24	0,07
46899000000,00	770625,38	295,00	8,40	2	0,06
1795600,00	758343,94	138,00	6,80	4	0,08
1004600,00	782033,65	97,00	5,20	16	0,14
1367400,00	807137,19	141,00	7,00	10	0,09
46476000000,00	759596,99	105,00	6,10	11	0,08
3099900,00	824667,79	126,00	7,30	14	0,09
37614000000,00	835804,43	155,00	7,50	26	0,06
1529800,00	641156,33	155,00	8,00	16,00	0,08
1105600,00	766506,51	106,00	7,00	19,00	0,08
48084000000,00	433619,03	116,00	5,90	28,00	0,1
1225900,00	450897,59	160,00	7,10	27,00	0,1
1811900,00	474425,48	152,00	7,80	29,00	0,09
36194000000,00	454769,87	255,00	9,60	23,00	0,08
1637600,00	751639,90	147,00	7,00	26	0,1
35312000000,00	700380,71	134,00	7,50	24	0,07
2473100,00	757034,99	139,00	8,50	25	0,1

451024,44	380558,47	83,00	6,70	19	0,07
2978500,00	941035,67	133,00	8,00	18	0,1
3226500,00	611507,57	101,00	6,50	23	0,12
1350200,00	639625,08	107,00	6,30	20	0,08
2446000,00	717750,91	142,00	7,30	14	0,1
1697300,00	752518,33	228,00	7,70	26	0,11
799148,57	678366,66	165,00	7,50	24	0,09
	1575000,00	106,00	6,90	19	0,07
		142,00	7,30	18	0,13
1410700,00	1099700,00	125,00	6,30	7,00	0,08
2955300,00	553702,48	129,00	5,50	2,00	0,01
53275000000,00	663928,57	113,00	5,90	24,00	0,07
2317400,00	549475,22	135,00	6,60	27,00	0,1
1143800,00	421284,12	182,00	8,20	25,00	0,09
53849000000,00	367112,18	148,00	6,80	21	0,1
1669800,00	537468,53	123,00	5,60	27	0,06
1947000,00	672000,00	259,00	10,90	6	0,09
1724700,00	724712,35	86,00	5,70	30	0,07
2486700,00	867772,29	173,00	6,90	25	0,08
2665900,00	1912900,00	121,00	6,00	24	0,06
1458600,00	1068900,00	90,00	5,90	30	0,08
42873000000,00	678312,21	143,00	7,20	4	0,12
1163700,00	541892,79	229,00	7,80	28	0,95
1493900,00	741978,21	126,00	6,10	20	0,08
969588,42	345508,99	152,00	7,90	27,00	0,07
34930000000,00	778627,48	86,00	5,20	28,00	0,13
53487000000,00	957713,44	112,00	5,30	20,00	0,07
1091400,00	706779,64	119,00	6,70	12,00	0,08
1882900,00	718327,19	178,00	6,60	29,00	0,08
2543600,00	551706,02	184,00	7,20	29	0,09
1667000,00	816326,21	141,00	7,50	14	
1416800,00	1056700,00	159,00	7,30	7	0,11
1058100,00	852323,89	109,00	6,00	30	0,12
1199600,00	1392000,00	111,00	6,10	26	0,08
1783100,00	752206,94	122,00	6,60	30	0,08
1028400,00	789564,02	109,00	5,50	17	0,14
3045300,00	711394,70	154,00	8,20	16	0,1
38906000000,00	943196,87	155,00	6,80	27	0,07
1226400,00	718762,07	196,00	8,80	14,00	0,08
1041900,00	571496,97	108,00	6,30	29,00	0,08
51830000000,00	373156,93	123,00	6,00	25,00	0,12
1306200,00	477078,52	151,00	7,20	27,00	
38488000000,00	448430,20	157,00	8,60	21,00	0,09
1419900,00	683677,99	105,00	6,20	27	0,09
35595000000,00	644223,25	128,00	7,00	30	0,06
2300900,00	758230,08	134,00	7,40	30	0,01
307486,80	322150,72	115,00	6,30	7	0,07
33335000000,00	1083300,00	125,00	6,80	18	0,08
36108000000,00	475124,29	134,00	6,90	22	0,12

1440200,00	549587,72	135,00	6,50	22	0,07
2394700,00	795924,71	136,00	6,90	17	0,1
1199900,00	942685,99	143,00	7,30	26	0,1
949674,54	608598,15	256,00	8,50	20	0,09
1345500,00	595274,87	135,00	6,40	21	
1496500,00	1004000,00	104,00	6,90	7,00	0,01
2724500,00	619035,15	114,00	5,50	2,00	0,1
53212000000,00	634254,20	106,00	6,10	1,00	0,01
2131700,00	573883,60	185,00	7,50		0,09
1439300,00	673858,94	74,00	6,40	0,00	0,09
56446000000,00	387156,58	133,00	6,80	10	0,1
1782200,00	601601,91	116,00	6,00	0	0,06
1946600,00	571684,71	276,00	11,20		0,12
2081600,00	1040900,00	111,00	5,40	6	0,06
2549800,00	803789,89	132,00	6,70	22	0,08
3089400,00	2501000,00	135,00	6,10	0	0,07
1440200,00	1072900,00	138,00	6,70	2	0,08
40805000000,00	642717,99	184,00	7,70	0	0,12
1393900,00	417892,34	237,00	9,20	8	0,08
1305500,00	718957,02	146,00	6,80		0,07
49833000000,00	492795,40	140,00	8,60	2,00	0,09
36090000000,00	667462,14	72,00	5,50	1,00	0,12
45310000000,00	985837,68	96,00	5,40	2,00	0,09
1010700,00	928655,16	106,00	6,40	5,00	0,09
1859200,00	738024,29	170,00	7,60	5,00	0,08
2751700,00	614607,36	179,00	7,30	4	0,09
1935800,00	806295,95		9,00	2	
1496900,00	1034400,00	156,00	6,70	3	0,11
1377200,00	980587,23	99,00	6,50		0,13
1152900,00	1358700,00	102,00	6,40	0	0,09
1792100,00	797188,42	165,00	6,40	0	0,07
1058200,00	773158,87		5,30	1	0,14
36935000000,00	705712,82	154,00	8,20	0	0,12
		192,00	7,40	22	0,06
1237600,00	959423,19	63,00	5,50	0,00	0,08
1078200,00	500736,21	102,00	6,60		0,08
50486000000,00	400914,93	92,00	5,60	0,00	0,12
1218500,00	561133,49	162,00	7,20	9,00	
38977000000,00	509916,91	195,00	8,20	9,00	0,08
1443000,00	560932,96	132,00	6,60	3	0,09
3178900,00	633988,71	168,00	8,10	1	0,06
2538500,00	733937,55	160,00	8,30	0	0,11
446101,94	240749,61	115,00	6,70	2	0,09
2785100,00	867379,61	156,00	7,00	0	0,09
37340000000,00	540946,07	126,00	6,80	0	0,1
1561100,00	510806,64	146,00	6,90	2	0,07
2342900,00	568014,84	130,00	6,80	13	0,09
1374800,00	790455,64	200,00	6,90	13	0,09
917623,54	676231,61	197,00	8,60	2	0,09
1390700,00	671713,14	129,00	6,50		

IMTSX (cm)	FMD %	timeperiod	Età	male	cigarettes	Fumo sì (1) no (0)
0,09	4,4	10	42	0	50	1
0,07	16,9	10	51	0	50	1
0,1	2,2	10	61	0	20	1
0,1	7,3	10	59	0	20	1
0,08	10,8	10	55	0	40	1
0,11	4	10	57	0	30	1
0,07	17,5	10	57	0	50	1
0,1	5	10	48	0	40	1
0,1	4,3	10	54	0	0	0
0,1	11,6	10	50	0	40	1
0,1	10,5	10	54	0	40	1
0,14	6,5	10		0	0	0
0,07	9,3	10	56	0	0	0
0,1	9,5	10	63	0	0	0
0,11	3,2	10	60	0	0	0
0,07	14,2	10	56	0	0	0
0,1	5,9	10	49	0	0	0
0,1	7,8	10	59	0	0	0
0,1	5	10	51	0	0	0
0,11	9,8	10	49	0	0	0
0,07	7,8	10	52	0	40	1
0,08	11,8	10	63	0	0	0
0,09	5,4	10	60	0	10	1
0,08	8,8	10	56	0	40	1
0,1	1,2	10	56	0	30	1
0,08	10,2	10	55	0	0	0
0,12	6	10	64	0	40	1
0,07	6,9	10	62	0	30	1
0,07	8,2	10	64	0	20	1
0,11	10,5	10	56	0	20	1
0,11	8,3	10	58	0	0	0
0,08	7,4	10	47	0	10	1
0,14	9	10	58	0	0	0
0,14	8,8	10	61	0	0	0
0,07	4,1	10	59	0	0	0
0,1	8,6	10	55	0	30	1
0,1	4,9	10	49	0	0	0
0,09	4,4	10	49	0	0	0
0,09	9,7	10	51	0	0	0
0,09	9	10	52	0	0	0
0,08		10	53	0	40	1
0,08	5,8	10	55	0	0	0
0,08	9,2	10	61	0	0	0
0,1	2,1	10	54	0	0	0
0,06	3,4	10	55	0	0	0

0,1	25,8	10	56	0	0	0
0,12	5,2	10	54	0	20	1
0,07	6,2	10	60	0	40	1
0,12	1,4	10	55	0	0	0
0,1	7,8	10	59	0	20	1
0,09	2,2	10	60	0	0	0
0,08	4,1	10	59	0	20	1
0,11	5,1	10	53	0	50	1
0,08	2	10	44	0	0	0
0,09	19,11	10	51	0	0	0
0,07	4,4	10	61	0	20	1
0,13	7,5	10	59	0	20	1
0,11	11	10	55	0	20	1
0,08	4,2	10	57	0	30	1
0,12	5,1	10	48	0	20	1
0,08	9,5	10	54	0	0	0
0,1	7,2	10	50	0	20	1
0,08	12,5	10	54	0	20	1
0,13	5,5	10	56	0	20	1
0,06	12	10	63	0	0	0
0,09	6	10	60	0	0	0
0,12	2,38	10	56	0	0	0
0,07	7,6	10	49	0	0	0
0,13	7	10	59	0	0	0
0,11	8,3	10	51	0	0	0
0,11	5,5	10	52	0	20	1
0,07	13,5	10	63	0	0	0
0,08	5	10	60	0	20	1
0,09	6,94	10	56	0	40	1
0,08	9,5	10	56	0	30	1
		10	55	0	0	0
0,08	12,5	10	64	0	20	1
0,11	8	10	62	0	30	1
0,08	5,6	10	64	0	20	1
0,08	3,9	10	58	0	0	0
0,13	6,7	10	47	0	20	1
0,09	5,5	10	59	0	0	0
0,08	4,4	10	55	0	30	1
0,1	1,9	10	49	0	0	0
0,1	6,2	10	49	0	0	0
0,09	9,7	10	51	0	0	0
		10	52	0	0	0
0,08	2,6	10	55	0	0	0
0,07	10,7	10	61	0	0	0
0,08	5,7	10	54	0	0	0
0,01	8,3	10	55	0	0	0
0,07	12,16	10	56	0	0	0
0,1	4,5	10	54	0	20	1
0,12	3,3	10	60	0	20	1

0,07	11	10	55	0	0	0
0,12	10	10	59	0	20	1
0,1	5,2	10	60	0	0	0
0,07	6,5	10	59	0	20	1
		10	53	0	20	1
0,09	8,3	10	51	0	0	0
0,09	7,6	10	61	0	20	1
0,13	18,4	10	59	0	40	1
0,09	6,9	10	55	0	30	1
1,8	4,2	10	57	0	30	1
0,09	10	10	48	0	50	1
0,08	5,4	10	54	0	0	0
0,1	10,5	10	50	0	50	1
0,08	13,5	10	54	0	30	1
0,13	5,5	10	56	0	40	1
0,08	3,4	10	63	0	0	0
0,1	6,9	10	60	0	0	0
0,1	7,6	10	56	0	0	0
0,75	2,3	10	49	0	0	0
0,09	7	10	59	0	0	0
0,09	10,5	10	51	0	0	0
0,1	7,3	10	52	0	50	1
0,07	10,5	10	63	0	0	0
0,07	3,3	10	60	0	20	1
0,09	6,9	10	56	0	40	1
0,13	2,3	10	56	0	30	1
		10	55	0	0	0
0,09	11,7	10	64	0	20	1
0,13	10	10	62	0	30	1
0,08	5,8	10	64	0	20	1
0,14	2	10	58	0	0	0
0,13	6,7	10	47	0	20	1
0,12	5,4	10	59	0	0	0
0,08	2,2	10	55	0	30	1
0,1	2	10	49	0	0	0
0,1	6,6	10	49	0	0	0
0,1	9,7	10	51	0	0	0
		10	52	0	0	0
0,07	0	10	55	0	0	0
0,08	5,9	10	61	0	0	0
0,09	15,2	10	54	0	0	0
0,11	5,9	10	55	0	0	0
0,1	13,6	10	56	0	0	0
0,1	2,2	10	54	0	20	1
0,12	2,2	10	60	0	30	1
0,08	4,4	10	55	0	0	0
0,1	2,4	10	59	0	20	1
0,09	2	10	60	0	0	0
0,07	2,9	10	59	0	20	1
		10	53	0	50	1

family_hx	diabetes	lvh	simd	Systolic pressure	Diastolyci pressure	CT 1
0	1	0	20	110	80	197,00
0	1	0	20	110	70	201,00
0	1	0	20	140	80	214,00
0	1	0	20	120	70	218,00
0	1	0	20	110	70	147,00
0	1	0	20	140	90	141,00
0	1	0	20	120	80	149,00
0	1	0	20	110	60	220,00
0	1	0	20	130	90	209,00
0	1	0	20	110	80	195,00
0	1	0	20	110	80	147,00
0	1	0	20	140	90	226,00
0	1	0	20	120	80	221,00
0	1	0	20	160	80	165,00
0	1	0	20	120	80	228,00
0	1	0	20	120	90	175,00
0	1	0	20	110	70	158,00
0	1	0	20	130	90	252,00
0	1	0	20	130	90	208,00
0	1	0	20	130	85	222,00
0	1	0	20	130	80	97,00
0	1	0	20	140	80	157,00
0	1	0	20	120	80	236,00
0	1	0	20	130	80	186,00
0	1	0	20	130	80	226,00
0	1	0	20	180	95	147,00
0	1	0	20	160	100	211,00
0	1	0	20	170	90	158,00
0	1	0	20	150	90	127,00
0	1	0	20	150	90	211,00
0	1	0	20	120	80	176,00
0	1	0	20	140	85	188,00
0	1	0	20	100	70	166,00
0	1	0	20	130	80	189,00
0	1	0	20	120	70	183,00
0	1	0	20	130	70	132,00
0	1	0	20	130	80	163,00
0	1	0	20	110	70	125,00
0	1	0	20	130	80	146,00
0	1	0	20	140	80	207,00
0	1	0	20	120	70	129,00
0	1	0	20	110	70	178,00
0	1	0	20	110	70	118,00
0	1	0	20	110	70	153,00
0	1	0	20	130	90	204,00

0	1	0	20	130	80	128,00
0	1	0	20	120	70	158,00
0	1	0	20	120	80	170,00
0	1	0	20	120	80	104,00
0	1	0	20	145	85	161,00
0	1	0	20	140	80	154,00
0	1	0	20	140	80	180,00
0	1	0	20	125	70	137,00
0	1	0	20	110	80	180,00
0	1	0	20	110	70	178,00
0	1	0	20	130	70	234,00
0	1	0	20	120	80	150,00
0	1	0	20	130	80	178,00
0	1	0	20	130	90	144,00
0	1	0	20	110	70	227,00
0	1	0	20	130	80	237,00
0	1	0	20	120	80	171,00
0	1	0	20	110	70	153,00
0	1	0	20	110	80	212,00
0	1	0	20	120	70	160,00
0	1	0	20	110	70	218,00
0	1	0	20	120	70	174,00
0	1	0	20	120	70	165,00
0	1	0	20	160	90	166,00
0	1	0	20	130	80	179,00
0	1	0	20	130	80	98,00
0	1	0	20	140	80	148,00
0	1	0	20	110	80	223,00
0	1	0	20	130	80	210,00
0	1	0	20	130	80	226,00
0	1	0	20	180	90	157,00
0	1	0	20	150	90	199,00
0	1	0	20	160	90	183,00
0	1	0	20	140	80	127,00
0	1	0	20	130	85	174,00
0	1	0	20	140	80	170,00
0	1	0	20	120	80	158,00
0	1	0	20	120	80	142,00
0	1	0	20	130	80	182,00
0	1	0	20	120	80	139,00
0	1	0	20	120	80	153,00
0	1	0	20	120	80	207,00
0	1	0	20	110	70	254,00
0	1	0	20	120	80	147,00
0	1	0	20	120	80	177,00
0	1	0	20	140	80	193,00
0	1	0	20	130	90	183,00
0	1	0	20	140	80	152,00
0	1	0	20	120	80	160,00

0	1	0	20	120	80	124,00
0	1	0	20	140	80	160,00
0	1	0	20	150	90	169,00
0	1	0	20	120	70	183,00
0	1	0	20	120	80	152,00
0	1	0	20	110	70	197,00
0	1	0	20	130	70	196,00
0	1	0	20	110	80	147,00
0	1	0	20	130	90	252,00
0	1	0	20	140	80	106,00
0	1	0	20	110	75	214,00
0	1	0	20	140	85	265,00
0	1	0	20	140	90	187,00
0	1	0	20	110	80	150,00
0	1	0	20	110	80	220,00
0	1	0	20	160	85	164,00
0	1	0	20	130	85	205,00
0	1	0	20	120	75	177,00
0	1	0	20	120	70	147,00
0	1	0	20	140	90	169,00
0	1	0	20	125	90	201,00
0	1	0	20	125	80	108,00
0	1	0	20	130	70	164,00
0	1	0	20	120	75	234,00
0	1	0	20	130	80	103,00
0	1	0	20	120	85	229,00
0	1	0	20	150	100	180,00
0	1	0	20	140	90	215,00
0	1	0	20	120	80	186,00
0	1	0	20	150	90	140,00
0	1	0	20	140	90	197,00
0	1	0	20	130	80	182,00
0	1	0	20	130	80	158,00
0	1	0	20	130	80	142,00
0	1	0	20	130	90	149,00
0	1	0	20	110	70	118,00
0	1	0	20	120	80	183,00
0	1	0	20	120	80	198,00
0	1	0	20	120	80	115,00
0	1	0	20	110	80	196,00
0	1	0	20	120	70	167,00
0	1	0	20	130	85	219,00
0	1	0	20	140	80	139,00
0	1	0	20	120	80	156,00
0	1	0	20	120	80	159,00
0	1	0	20	120	80	107,00
0	1	0	20	150	80	162,00
0	1	0	20	140	80	173,00
0	1	0	20	140	95	161,00
0	1	0	20	110	80	154,00

HDL 1	CHD	CHD corrected	CHD change	MI change	STROKE change
34,00	10,516	0,053			
42,00	14,648	0,073			
30,00	34,894	0,163			
41,00	22,909	0,105			
26,00	19,997	0,136			
39,00	18,286	0,130			
44,00	13,782	0,092			
37,00	16,190	0,074			
41,00	14,504	0,069			
52,00	10,460	0,054			
35,00	14,173	0,096			
39,00					
39,00	15,501	0,070			
49,00	15,136	0,092			
41,00	16,681	0,073			
47,00	9,367	0,054			
42,00	5,840	0,037			
41,00	20,096	0,080			
50,00	10,064	0,048			
31,00	17,213	0,078			
37,00	9,557	0,099			
43,00	13,697	0,087			
45,00	23,017	0,098			
34,00	24,225	0,130			
44,00	22,890	0,101			
30,00	21,940	0,149			
48,00	28,461	0,135			
32,00	32,355	0,205			
34,00	23,082	0,182			
41,00	26,870	0,127			
52,00	8,790	0,050			
52,00	12,341	0,066			
53,00	5,676	0,034			
42,00	15,083	0,080			
66,00	6,870	0,038			
56,00	9,404	0,071			
51,00	6,280	0,039			
44,00	3,760	0,030			
45,00	7,207	0,049			
41,00	14,824	0,072			
37,00	12,518	0,097			
45,00	8,460	0,048			
56,00	4,035	0,034			
46,00	6,371	0,042			
54,00	10,506	0,051			

47,00	7,025	0,055			
28,00	21,585	0,137			
43,00	17,474	0,103			
30,00	8,218	0,079			
36,00	24,336	0,151			
55,00	9,338	0,061			
39,00	24,056	0,134			
37,00	14,254	0,104			
48,00	3,879	0,022			
37,00	9,230	0,052	5,351	2,411	0,493
34,00	31,784	0,136	22,554	18,820	6,424
42,00	15,360	0,102	-16,424	-12,583	-2,676
35,00	22,130	0,124	6,770	5,695	0,294
39,00	16,951	0,118	-5,179	-4,102	0,640
47,00	12,811	0,056	-4,141	-2,928	-4,144
54,00	12,190	0,051	-0,620	-2,968	0,980
49,00	10,978	0,064	-1,213	1,589	0,025
29,00	18,126	0,118	7,148	4,765	-0,090
40,00	19,263	0,091	1,137	0,699	0,368
58,00	7,481	0,047	-11,781	-10,077	0,425
52,00	10,580	0,049	3,099	1,507	-1,317
51,00	8,339	0,048	-2,241	-0,958	0,152
47,00	6,209	0,038	-2,130	-0,850	-0,862
46,00	15,198	0,092	8,989	5,611	6,507
52,00	7,827	0,044	-7,371	-4,708	-5,727
32,00	11,622	0,119	3,795	4,566	2,024
42,00	13,145	0,089	1,523	-1,634	1,907
60,00	14,502	0,065	1,356	2,827	-2,313
44,00	21,379	0,102	6,878	6,010	1,758
59,00	17,169	0,076	-4,211	-3,223	-0,042
40,00	17,633	0,112	0,464	-1,584	3,994
51,00	24,006	0,121	6,374	7,142	5,185
40,00	28,772	0,157	4,765	3,941	1,926
38,00	19,092	0,150	-9,679	-8,001	-5,162
56,00	8,980	0,052	-10,112	-9,657	-8,009
65,00	8,163	0,048	-0,817	2,135	0,388
45,00	9,481	0,060	1,318	-2,029	-1,103
61,00	8,075	0,057	-1,406	1,002	1,049
51,00	7,353	0,040	-0,721	-1,650	-1,891
43,00	5,491	0,040	-1,863	-1,119	-0,494
44,00	6,884	0,045	1,393	0,632	0,221
44,00	10,824	0,052	3,940	2,167	0,135
52,00	11,110	0,044	0,286	-0,181	-0,201
73,00	4,480	0,030	-6,631	-3,326	1,470
56,00	6,941	0,039	2,462	1,258	-1,041
60,00	9,648	0,050	2,706	1,732	1,778
53,00	9,691	0,053	0,043	-0,162	-0,707
30,00	23,411	0,154	13,719	12,977	3,375
40,00	17,687	0,111	-5,723	-5,383	-1,173

34,00	8,798	0,071	-8,889	-8,212	-3,127
35,00	23,852	0,149	15,054	13,572	6,611
61,00	10,311	0,061	-13,541	-12,394	-2,167
36,00	21,978	0,120	11,667	10,298	-1,580
37,00	15,109	0,099	-6,869	-4,802	-1,612
48,00	7,443	0,038	-7,666	-7,378	-2,203
39,00	24,639	0,126	17,197	14,462	6,350
47,00	11,588	0,079	-13,052	-10,321	-3,970
32,00	31,877	0,126	20,289	16,082	1,747
35,00	15,150	0,143	-16,727	-12,540	2,335
43,00	13,268	0,062	-1,882	-1,461	-5,916
58,00	14,255	0,054	0,987	-1,817	1,841
53,00	14,039	0,075	-0,216	2,998	1,269
34,00	14,977	0,100	0,938	-0,513	-2,213
43,00	18,592	0,085	3,615	2,561	0,381
52,00	14,064	0,086	-4,528	-5,088	6,893
42,00	16,176	0,079	2,111	0,786	-5,940
58,00	7,147	0,040	-9,028	-5,347	-1,698
43,00	5,970	0,041	-1,177	-0,353	-0,855
53,00	10,763	0,064	4,793	2,572	3,427
62,00	6,706	0,033	-4,057	-2,221	-2,947
41,00	9,009	0,083	2,303	3,127	1,737
48,00	11,317	0,069	2,308	-0,803	1,030
52,00	19,916	0,085	8,599	8,468	0,628
40,00	10,891	0,106	-9,025	-6,373	0,165
54,00	17,170	0,075	6,279	4,498	-1,322
25,00	25,185	0,140	8,014	3,308	0,871
52,00	23,376	0,109	-1,809	1,765	6,550
39,00	21,729	0,117	-1,647	-1,891	-5,602
38,00	22,794	0,163	1,065	1,755	8,729
53,00	12,647	0,064	-10,147	-10,458	-10,100
56,00	9,614	0,053	-3,033	0,784	-1,631
45,00	10,793	0,068	1,179	-2,105	0,746
56,00	10,330	0,073	-0,463	1,970	1,420
54,00	5,035	0,034	-5,295	-4,782	-3,227
41,00	3,841	0,033	-1,194	-0,714	-0,869
48,00	7,832	0,043	3,991	1,997	0,618
46,00	9,668	0,049	1,835	0,980	0,125
49,00	4,641	0,040	-5,027	-2,697	0,346
66,00	6,813	0,035	2,172	0,815	0,120
46,00	8,412	0,050	1,599	1,087	-0,224
51,00	12,295	0,056	3,883	2,381	0,892
49,00	8,466	0,061	-3,829	-2,148	1,109
30,00	19,955	0,128	11,490	10,225	-0,350
34,00	20,674	0,130	0,719	0,174	1,640
29,00	8,935	0,084	-11,739	-10,382	-3,155
33,00	27,337	0,169	18,402	16,459	9,095
68,00	8,218	0,048	-19,120	-16,702	-6,138
37,00	22,811	0,142	14,594	12,948	3,638
42,00	11,645	0,076	-11,166	-9,003	-6,289

CVD change	ASSIGN change	CUORE change	MI	STROKE	CVD
			7,794	1,060	16,394
			9,663	2,128	21,381
			25,182	9,946	50,137
			15,627	4,987	34,363
			13,323	2,811	27,680
			12,860	7,673	32,843
			8,851	4,342	24,408
			11,236	1,722	22,191
			7,098	2,704	20,315
			6,594	1,965	16,978
			9,037	2,605	21,795
			7,379	2,293	20,272
			7,812	9,904	29,606
			7,969	2,939	22,967
			3,731	2,260	14,445
			2,023	1,004	8,372
			10,482	3,742	27,457
			4,535	2,185	15,241
			9,346	1,926	21,459
			6,193	4,171	19,632
			6,499	6,108	24,875
			15,667	5,292	34,991
			17,156	5,572	36,194
			16,143	5,560	34,960
			13,236	9,569	35,493
			21,008	18,428	50,039
			24,242	20,483	53,768
			16,622	14,661	43,343
			19,825	9,375	42,421
			3,363	2,556	14,455
			9,182	3,868	23,373
			1,605	1,285	8,638
			7,183	4,171	23,659
			2,354	2,700	12,640
			5,893	5,077	20,351
			2,469	1,873	10,922
			1,062	0,994	6,230
			2,878	2,166	12,258
			7,672	3,113	21,558
			8,149	3,349	21,622
			3,121	1,530	11,929
			0,999	2,177	8,551
			2,102	1,423	9,655
			4,582	2,859	16,706

			2,602	3,016	13,186
			14,973	3,641	30,870
			11,504	5,241	29,628
			3,138	2,112	12,995
			17,552	9,898	40,693
			3,915	5,090	18,564
			17,179	8,729	39,491
			9,613	3,907	24,343
			1,302	0,679	6,112
5,695	57,524	1,804	3,713	1,171	11,807
33,705	42,333	30,504	22,534	7,596	45,512
-18,551	-99,491	-18,438	9,950	4,919	26,961
6,849	99,434	1,277	15,645	5,213	33,810
-4,194	-98,349	-1,550	11,543	5,853	29,617
-10,628	88,234	-9,753	8,615	1,710	18,989
-0,890	-73,279	1,084	5,647	2,690	18,098
1,089	-16,472	-1,517	7,236	2,715	19,187
6,392	99,795	6,360	12,001	2,625	25,580
1,828	0,128	4,689	12,700	2,993	27,408
-12,675	-100,000	-5,005	2,623	3,418	14,733
0,745	5,955	-3,773	4,130	2,101	15,478
-2,092	-5,922	0,492	3,172	2,253	13,386
-3,597	0,026	-5,130	2,321	1,391	9,789
17,811	0,312	13,658	7,932	7,897	27,600
-14,671	-0,343	-10,161	3,225	2,170	12,929
9,114	0,094	-0,780	7,790	4,194	22,044
2,231	0,256	4,727	6,156	6,101	24,274
0,255	-0,149	1,098	8,983	3,788	24,529
9,009	99,286	8,058	14,993	5,546	33,538
-4,138	-98,802	-1,670	11,770	5,504	29,401
1,834	2,367	-1,573	10,186	9,498	31,235
12,993	0,234	19,506	17,329	14,682	44,228
4,862	89,175	0,717	21,269	16,609	49,090
-11,589	-92,036	-10,676	13,269	11,446	37,501
-21,466	-0,450	-19,654	3,611	3,437	16,035
2,323	-0,003	-1,756	5,747	3,825	18,358
-2,839	0,153	5,438	3,717	2,722	15,518
1,611	-0,153	-0,901	4,719	3,772	17,129
-5,032	0,062	-3,253	3,069	1,880	12,098
-3,072	-0,041	-1,397	1,951	1,387	9,026
1,749	0,059	1,064	2,583	1,607	10,775
4,056	56,090	-0,457	4,749	1,743	14,831
-0,441	42,290	1,533	4,569	1,541	14,390
-4,170	-98,459	-1,784	1,242	3,011	10,220
1,191	0,003	-0,569	2,500	1,970	11,412
5,736	0,000	1,867	4,232	3,748	17,148
-0,989	0,035	-0,133	4,070	3,041	16,159
20,361	98,976	4,580	17,047	6,416	36,520
-6,677	-93,207	7,855	11,664	5,243	29,843

-16,252	-5,101	-13,987	3,452	2,116	13,591
25,708	66,767	18,533	17,024	8,726	39,300
-18,186	-67,473	-14,405	4,630	6,560	21,114
12,381	99,994	11,832	14,928	4,979	33,495
-9,192	-93,259	-13,341	10,126	3,368	24,303
-14,134	-4,684	-3,316	2,748	1,165	10,169
28,995	97,948	23,219	17,209	7,515	39,163
-18,188	-99,966	-13,876	6,889	3,545	20,975
21,516	99,967	13,219	22,970	5,292	42,491
-12,970	-99,867	-11,308	10,430	7,627	29,521
-10,088	99,858	-9,253	8,969	1,712	19,433
2,182	-46,782	3,062	7,153	3,552	21,615
4,217	-53,030	-1,515	10,151	4,822	25,831
-3,246	30,488	3,643	9,638	2,609	22,585
4,198	69,335	5,706	12,199	2,990	26,783
1,628	-99,987	3,652	7,110	9,882	28,411
-4,151	97,894	-9,917	7,897	3,943	24,260
-12,149	-97,906	-2,707	2,550	2,244	12,111
-2,572	0,055	-4,741	2,197	1,389	9,539
10,268	-0,047	9,405	4,769	4,816	19,806
-8,658	-0,007	-6,131	2,547	1,869	11,148
7,055	0,005	-1,035	5,674	3,606	18,203
2,458	0,085	4,314	4,871	4,636	20,660
11,386	59,301	5,865	13,339	5,264	32,047
-9,524	-59,387	-3,866	6,967	5,430	22,523
4,949	14,245	5,791	11,465	4,107	27,471
6,278	85,749	-1,657	14,773	4,978	33,749
8,016	-87,575	19,017	16,538	11,527	41,765
-6,873	86,258	-10,175	14,646	5,926	34,891
8,173	-96,083	6,501	16,402	14,655	43,064
-21,616	-2,311	-22,468	5,944	4,555	21,449
-2,738	-0,283	-3,638	6,728	2,924	18,711
-0,331	0,165	6,470	4,622	3,670	18,380
3,107	-0,170	-0,364	6,593	5,090	21,487
-12,003	0,000	-6,279	1,810	1,863	9,484
-3,165	0,004	-1,090	1,096	0,995	6,318
5,423	0,384	2,668	3,094	1,612	11,741
1,993	7,848	-1,551	4,074	1,737	13,734
-4,740	-8,235	-1,205	1,376	2,083	8,995
2,904	0,000	2,662	2,191	2,204	11,898
1,048	0,192	-1,607	3,277	1,980	12,947
5,562	9,790	2,467	5,659	2,872	18,508
-2,398	-9,981	-1,480	3,511	3,981	16,111
13,246	99,798	2,924	13,735	3,631	29,357
3,422	-13,722	11,189	13,909	5,271	32,779
-19,048	-84,758	-15,367	3,527	2,117	13,730
30,632	97,951	22,268	19,985	11,212	44,362
-27,160	-99,271	-19,446	3,283	5,074	17,202
21,112	34,132	15,468	16,232	8,712	38,314
-19,383	-33,016	-16,647	7,229	2,423	18,931

ASSIGN	Progetto Cuore	BMI	Compliance	Terapia IGO sì(1) no(0)
100,000	3,676155	25,4		0
97,843	6,622493	24,5	95,7	0
100,000	23,92501	25,4		0
100,000	23,08474	19,4		0
100,000	12,6266	19,8	96,5	0
1,213	17,2272	19,8		0
0,288	13,35284	23,9		0
100,000	10,10227	24,6	92,2	0
99,932	6,889888	24,6		0
0,475	5,237063	23,2		0
17,127	10,53189	30,1		0
	0,152403	28,0	76,8	0
100,000	7,7335	30,1		0
0,082	21,02733	30,9		0
100,000	10,49214	30,9	85,1	0
0,351	8,593004	31,2		0
0,358	2,699015	29,4		0
100,000	19,79219	29,1	83,8	0
3,344	7,875841	28,7		0
100,000	9,345366	19,5		0
0,011	5,33479	20,5	96,7	0
0,679	10,85508	19,8		0
100,000	16,76339	29,7		0
100,000	19,35424	30,4	75,3	0
100,000	21,34632	29,7		0
90,295	17,43173	29,1		0
79,563	43,17348	27,8		1
99,997	39,05991	28,4		1
4,596	30,20769	28,4	96,4	1
100,000	25,61601	28,4		1
0,028	5,861961	36,7		1
0,096	5,884751	29,4	96,4	1
0,004	4,237464	29,4		1
41,896	10,0927	32,7		1
0,000	8,834342	32,4	92,9	1
0,000	10,26627	32,4		1
0,006	5,171202	25,6		1
0,002	3,506926	26,2	56,3	1
0,021	5,856277	27,5		1
99,570	6,667427	30,6		1
0,643	6,09966	24,5		1
1,308	4,57324	32,5		1
0,000	4,367926	33,9	74,6	1
0,029	3,618874	35,1		1
0,292	6,117414	20,3		1

0,001	4,618124	20,0	100,0	1
100,000	8,683798	20,6		1
6,660	18,68798	28,4		1
0,518	4,070755	28,1	100,0	1
53,681	24,071	27,7		1
0,000	7,399017	25,8		1
78,392	24,28085	25,3	87,3	1
2,440	6,803998	24,8		1
0,144	1,955562	23,2		1
57,667	3,760058	34,1		1
100,000	34,26405	33,4	94,6	1
0,509	15,82566	33,4		1
99,943	17,10241	30,2		1
1,594	15,55229	27,0		1
89,829	5,79973	26,4	92,2	1
16,550	6,883244	26,6		1
0,077	5,366018	31,3		1
99,872	11,72615	30,8	84,3	1
100,000	16,4154	30,8		1
0,000	11,41088	29,4		1
5,955	7,637531	29,0	95,8	1
0,033	8,129042	29,8		1
0,060	2,999211	35,8		1
0,371	16,65678	35,8	88,7	1
0,028	6,495714	36,8		1
0,122	5,715899	41,4		0
0,378	10,44264	28,7		0
0,229	11,54036	28,0	98,6	0
99,515	19,59844	28,0		0
0,713	17,92819	23,2		0
3,080	16,35555	23,2	98,8	0
3,314	35,86191	24,2		0
92,489	36,57899	25,8		0
0,452	25,90349	25,7	98,0	0
0,003	6,249147	25,7		0
0,000	4,493519	25,1		0
0,153	9,931317	25,7	85,7	0
0,000	9,030316	21,7		0
0,062	5,777259	25,2		0
0,020	4,380758	28,7		0
0,080	5,444866	29,4	53,7	0
56,170	4,98813	29,4		0
98,459	6,520881	33,0		0
0,000	4,736842	32,7	97,4	0
0,003	4,167545	33,0		0
0,003	6,034987	33,0		1
0,038	5,901899	34,3	75,4	1
99,014	10,48222	35,3		1
5,806	18,33766	30,4		1

0,706	4,350874	35,9		1
67,473	22,88361	37,1	100,0	1
0,000	8,478755	36,1		1
99,994	20,31102	24,5		1
6,735	6,970096	24,3	99,1	1
2,051	3,654571	24,1		1
99,999	26,87313	26,0		1
0,033	12,99714	26,6	74,6	1
100,000	26,21601	27,5		1
0,133	14,90807	35,5		1
99,990	5,655172	27,1		1
53,208	8,71704	28,2	100,0	1
0,178	7,202261	28,4		1
30,665	10,84484	32,7		1
100,000	16,55079	33,0	96,4	1
0,013	20,20272	32,5		1
97,907	10,28572	29,0		1
0,001	7,579042	28,6	93,4	1
0,056	2,838212	29,0		1
0,009	12,24292	32,2		1
0,002	6,111491	33,6	50,0	1
0,006	5,07639	31,2		1
0,091	9,389939	30,9		1
59,393	15,25512	31,2	98,0	1
0,006	11,38943	30,5		1
14,251	17,18031	21,7		1
100,000	15,52376	20,8	89,7	1
12,425	34,54063	21,4		1
98,683	24,36521	31,7		1
2,600	30,86574	30,8	94,3	1
0,289	8,398037	27,7		1
0,006	4,760012	26,0		0
0,171	11,22977	25,3	99,7	0
0,000	10,86532	26,6		0
0,000	4,58666	37,4		0
0,004	3,496606	38,8	77,0	0
0,388	6,165003	37,7		0
8,235	4,613616	36,7	97,2	1
0,000	3,408414	36,7		1
0,000	6,070181	29,0		1
0,192	4,462884	27,8	94,7	1
9,983	6,92988	28,7		1
0,001	5,450163	24,8	98,3	1
99,799	8,373965	24,6		1
86,078	19,56339	31,7		1
1,320	4,196198	32,9	69,0	1
99,271	26,46371	32,7		1
0,000	7,017299	28,1		1
34,132	22,48579	28,7	95,4	1
1,116	5,839257	28,4		1

Tp lipidi sì (1) no(0)	Tg 1	LDL1	GOT 1	GPT 1	FIBRINOG	Microalbu	RDW 1
0	87,00	59	22,00	32,00	95,00	4,00	13,00
0	69,00	85,2	21,00	24,00	106,00	9,00	13,70
0	72,00		24,00	34,00	118,00	4,50	12,90
0	173,00		22,00	19,00	320,00	3,00	13,10
0	142,00		26,00	22,00	298,00	2,20	12,90
0	143,00		21,00	17,00	285,00	2,00	13,70
0	88,00		25,00	16,00	245,00	5,20	12,70
0	80,00		18,00	16,00	219,00	3,00	13,30
0	104,00		21,00	20,00	221,00	4,00	12,60
1	266,00		18,00	22,00	233,00	57,60	13,80
1	399,00		18,00	22,00	331,00	1,00	12,80
1	304,00		17,00	17,00	281,00	1,00	12,70
1	112,00		31,00	30,00	282,00	1,00	13,10
1	103,00		20,00	18,00	365,00	2,00	13,10
1	151,00		15,00	13,00	289,00	3,00	13,30
0	131,00		18,00	14,00	249,00	2,00	16,20
0	226,00		37,00	70,00	351,00	5,00	13,60
0	152,00		44,00	82,00	347,00	3,00	13,30
0	160,00		33,00	56,00	307,00	9,00	13,70
1	120,00		20,00	19,00	259,00	3,00	17,80
1	150,00		19,00	21,00	239,00	1,00	17,20
1	91,00		28,00	34,00	229,00	3,00	16,50
0	41,00		13,00	10,00		16,00	14,20
0	57,00		16,00	15,00	285,00	22,00	14,40
0	85,00		14,00	11,00	258,00	30,00	14,30
1	118,00		68,00	13,00	377,00	7,00	15,10
1	176,00	127,8	17,00	16,00		13,00	12,50
1	148,00	95	15,00	18,00	402,00	12,00	13,50
0	172,00	118	18,00	20,00	255,00	22,00	13,70
0	106,00	60,4	11,00	16,00	284,00	10,00	13,90
0	227,00	50	11,00	19,00	331,00	10,00	14,30
0	129,00	104,2	13,00	18,00	320,00	11,00	13,30
0	240,00	78	16,00	17,00	282,00	5,00	
0	74,00	86	21,00	29,00	356,00	6,00	13,70
0	76,00	154,2	23,00	32,00	297,00	12,00	14,50
0	104,00	69,6	27,00	48,00	294,00	7,00	
0	105,00	97,4	22,00	20,00	250,00	11,00	13,30
0	74,00	45,4	24,00	21,00	386,00	14,00	13,20
0	108,00		25,00	38,00	249,00	3,00	12,60
0	193,00	80,8	65,00	86,00	221,00	10,00	13,40
0	115,00	62,2	21,00	20,00	341,00	34,00	13,10
1	89,00	109,6	20,00	24,00	254,00	26,00	13,70
1	103,00		18,00	32,00	240,00	35,00	13,50
1	116,00	98,8	24,00	43,00	229,00	53,00	13,10
1	78,00		14,00	11,00	305,00	192,00	14,80

1	95,00		13,00	7,00	347,00	199,70	15,60
1	69,00		15,00	12,00	343,00	105,00	14,60
0	95,00		16,00	18,00	273,00	5,00	12,60
0	99,00		15,00	11,00	230,00	1,80	13,20
0	104,00		14,00	13,00	259,00	3,00	12,50
1	81,00		21,00	20,00	287,00	9,10	13,40
1	56,00		20,00	17,00	309,00	1,00	13,70
1	91,00		19,00	18,00	398,00	2,00	13,40
0	64,00		14,00	13,00	240,00	1,20	12,70
0	240,00		24,00	36,00	293,00	2,00	12,90
0	236,00		23,00	36,00	301,00	4,00	13,40
0	122,00		21,00	35,00	241,00	3,00	12,60
1	70,00		18,00	23,00	300,00	3,00	13,90
1	141,00		26,00	29,00	243,00	12,00	13,60
1	115,00		22,00	17,00	216,00	22,00	13,90
1	143,00		26,00	16,00	231,00	18,00	14,20
0	105,00		19,00	36,00	284,00	3,00	13,30
0	67,00		16,00	22,00	277,00	3,00	14,20
0	69,00		16,00	20,00	271,00	6,00	13,60
0	134,00		24,00	41,00	296,00	3,00	12,50
0	117,00		25,00	43,00	249,00	2,00	13,40
0	172,00		30,00	59,00	278,00	3,00	12,10
1	132,00		15,00	22,00	292,00	3,00	13,60
1	161,00		13,00	32,00	273,00	3,00	14,50
1	120,00		14,00	18,00	307,00	3,00	13,80
0	87,00	59,8	18,00	14,00	317,00	4,00	14,90
0	120,00	153,2	36,00	30,00	183,00	9,00	12,60
0	162,00	134,8	35,00	27,00	172,00	12,00	13,20
0	188,00	62,2	29,00	24,00	175,00	6,00	
0	150,00	53,2	29,00	30,00	333,00	4,00	12,70
0	168,00	43,6	23,00	26,00	315,00	3,00	13,30
0	86,00	97,4	19,00	25,00	290,00	5,00	12,80
0	152,00		31,00	32,00	231,00	5,00	12,70
0	111,00		23,00	26,00	238,00	2,00	12,60
0	137,00		28,00	33,00	252,00	6,00	12,40
0	52,00		13,00	12,00	258,00	13,30	16,10
0	58,00		12,00	10,00	365,00	11,00	16,20
0	44,00		12,00	18,00	390,00	30,00	17,00
0	176,00		15,00	13,00	330,00	3,10	12,20
1	64,00		16,00	14,00	278,00	14,90	14,50
1	338,00		21,00	26,00	259,00	17,00	13,90
1	338,00		21,00	26,00	259,00	17,00	
0	126,00		24,00	14,00	300,00	11,00	13,60
0	145,00		25,00	16,00	302,00	17,00	13,10
0	138,00		34,00	21,00	270,00	11,00	13,40
0	119,00	60	15,00	19,00	402,00	12,00	13,70
0	163,00	154,8	15,00	19,00	320,00	13,00	14,00
0	414,00	93,8	16,00	22,00	330,00	19,00	13,90
0	229,00	98,2	13,00	15,00	334,00	6,00	13,60

1	168,00	107,6	26,00	29,00	250,00	6,00	13,00
1	228,00	123	27,00	30,00	237,00	9,00	12,80
1	215,00	74,4	28,00	34,00	295,00	10,00	12,60
1	90,00	152,2	18,00	17,00	336,00	7,00	13,60
1	68,00	65,6	25,00	24,00	392,00	7,00	14,00
1	77,00	141,2	18,00	17,00	318,00	7,00	14,30
1	216,00	88	24,00	34,00		41,00	12,30
1	257,00		19,00	24,00	193,00	15,20	13,40
1	226,00		18,00	24,00	229,00	8,00	12,70
0	206,00		28,00	51,00	265,00	33,00	14,00
0	206,00		20,00	20,00	287,00	10,00	13,00
0	128,00		19,00	31,00		1,60	12,40
0	229,00		31,00	31,00	282,00	5,00	13,40
0	107,00		22,00	20,00	412,00	19,50	16,80
0	134,00		20,00	18,00	351,00	16,00	14,20
0	169,00		28,00	23,00	413,00	18,00	16,10
1	121,00		16,00	16,00	379,00	4,00	14,50
1	118,00		17,00	22,00	319,00	3,00	14,80
1	74,00		22,00	23,00	278,00	3,00	14,50
1	210,00		17,00	16,00	284,00	4,00	13,30
1	180,00		22,00	28,00	226,00	7,00	13,40
1	172,00		24,00	19,00	215,00	9,00	13,40
1	142,00	95,2	19,00	25,00	286,00	6,00	14,10
1	99,00	127,6	28,00	51,00	287,00	4,00	13,30
1	61,00	63,8	23,00	35,00	258,00	8,00	13,50
1	69,00		14,00	11,00	322,00	58,00	15,20
1	77,00		16,00	21,00	251,00	2,00	14,30
1	70,00		14,00	13,00	196,00	2,00	14,70
1	114,00		24,00	50,00	275,00	12,00	14,30
1	126,00		24,00	44,00		8,00	14,20
1	83,00		18,00	21,00	286,00	6,00	13,90
0	96,00		11,00	7,00	402,00	27,00	14,10
0	78,00		10,00	7,00	463,00	20,00	13,70
0	110,00		12,00	8,00	361,00	34,00	14,20
0	102,00		28,00	31,00	421,00	19,00	12,60
0	255,00		24,00	39,00	326,00	21,00	12,70
0	265,00		32,00	42,00	347,00	11,00	13,10
0	470,00	146,2	26,00	28,00	265,00	3,00	14,00
0	126,00	75,6	17,00	19,00	226,00	20,00	13,90
1	259,00	86,2	29,00	34,00	358,00	9,00	14,80
1	125,00	115,8	31,00	34,00	428,00	5,00	14,40
1	174,00	99,8	29,00	40,00	372,00	11,00	14,60
0	129,00	138,6	21,00	18,00	324,00	9,00	13,30
0	111,00	73,6	21,00	20,00	322,00	4,10	14,20
0	186,00		15,00	15,00	397,00	40,60	13,70
0	113,00		16,00	15,00	318,00	345,00	14,90
0	185,00		16,00	19,00	362,00	69,00	14,20
0	155,00		15,00	18,00	253,00	9,00	13,50
0	166,00		19,00	23,00	283,00	13,60	13,60
0	85,00		18,00	20,00	219,00	15,00	13,00
